# BR-ENHANCED EXPRESSION 1/2/3 fine-tune plant photomorphogenesis by regulating the phyA/B-SPA1 cascade

**DOI:** 10.1093/plcell/koag284

**Published:** 2026-09-21

**Authors:** Jiale Yuan, Jing Cao, Qizhang Wang, Qian Xu, Yuxia Liang, Ruiyun Wang, Can Huang, Hua Zhou, Dongqing Xu, Fang Lin

**Affiliations:** Ministry of Education Key Laboratory of Cell Activities and Stress Adaptations, School of Life Sciences, Lanzhou University, Lanzhou 730000, China; Gansu Province Key Laboratory of Gene Editing Breeding, School of Life Sciences, Lanzhou University, Lanzhou 730000, China; Ministry of Education Key Laboratory of Cell Activities and Stress Adaptations, School of Life Sciences, Lanzhou University, Lanzhou 730000, China; Gansu Province Key Laboratory of Gene Editing Breeding, School of Life Sciences, Lanzhou University, Lanzhou 730000, China; Ministry of Education Key Laboratory of Cell Activities and Stress Adaptations, School of Life Sciences, Lanzhou University, Lanzhou 730000, China; Gansu Province Key Laboratory of Gene Editing Breeding, School of Life Sciences, Lanzhou University, Lanzhou 730000, China; State Key Laboratory of Microbial Technology, College of Life Sciences, Nanjing Normal University, Nanjing 210023, China; Ministry of Education Key Laboratory of Cell Activities and Stress Adaptations, School of Life Sciences, Lanzhou University, Lanzhou 730000, China; Ministry of Education Key Laboratory of Cell Activities and Stress Adaptations, School of Life Sciences, Lanzhou University, Lanzhou 730000, China; Ministry of Education Key Laboratory of Cell Activities and Stress Adaptations, School of Life Sciences, Lanzhou University, Lanzhou 730000, China; Gansu Province Key Laboratory of Gene Editing Breeding, School of Life Sciences, Lanzhou University, Lanzhou 730000, China; Laboratory of Plant and Environment Interaction Study, Tsientang Institute for Advanced Study, Hangzhou 310000, China; State Key Laboratory of Crop Genetics & Germplasm Enhancement and Utilization, National Center for Soybean Improvement, College of Agriculture, Nanjing Agricultural University, Nanjing 210095, China; Ministry of Education Key Laboratory of Cell Activities and Stress Adaptations, School of Life Sciences, Lanzhou University, Lanzhou 730000, China; Gansu Province Key Laboratory of Gene Editing Breeding, School of Life Sciences, Lanzhou University, Lanzhou 730000, China

## Abstract

BR-ENHANCED EXPRESSION1 (BEE1), BEE2, and BEE3, key transcriptional components of brassinosteroid signaling, have been shown to play critical roles in regulating light signaling. However, the precise molecular mechanism by which BEE1/2/3 repress photomorphogenesis remains elusive. In this study, we found that light triggers the degradation of BEE1/2/3 proteins through the 26S proteasome system. BEE1/2/3 interact with PHYTOCHROME A (phyA) and phyB, and this interaction promotes the degradation of BEE3. Mutations in *BEE1/2/3* exhibit short hypocotyls and partially suppress the long hypocotyl phenotype of the *phyA phyB* double mutant. BEE1/2/3 attenuate the interaction between phyB and PHYTOCHROME-INTERACTING FACTOR 3 (PIF3), leading to PIF3 stabilization and subsequent repression of photomorphogenesis. In addition, BEE1/2/3 interact with SUPPRESSORs OF PHYTOCHROME A (SPAs) to disrupt the phyA/B-SPA1 association, thereby further attenuating the exaggerated light signaling. The COP1–SPAs complexes stabilize BEE1/3 under both dark and light conditions. Mutations in *BEE1/2/3* enhance the constitutively photomorphogenic phenotype of *spa1/2/4*, whereas overexpression of *BEE1/3* partially rescue the short hypocotyls of *cop1* and *spa1/2/4*. Collectively, these findings demonstrate that BEE1/2/3 fine-tune the phyA/B-COP1/SPA1 signaling hub to negatively regulate photomorphogenesis in response to excessive light signaling. Our study provides insights into the pivotal roles of BEE1/2/3 as photomorphogenic repressors, facilitating adaptation to complex light conditions.

## Introduction

Light serves as an essential environmental cue for regulating plant growth and development throughout the plant life cycle (Jiao et al. 2007; Kami et al. 2010). Plants have evolved various photoreceptors to respond to different light wavelengths. PHYTOCHROME A–E (phyA-E) absorb red/far-red light (600 to 750 nm) (Li et al. 2011; Xu et al. 2015; Cheng et al. 2021). UV-A/blue light (350 to 500 nm) is monitored by CRYPTOCHROMES (CRYs), phototropins, and ZEITLUPE/FLAVIN-BINDING KELCH REPEAT F-BOX 1/LOV KELCH PROTEIN 2 (ZTL/FKF1/LKP2) family members (Wang and Lin 2020). UV-B light (275 to 320 nm) is sensed by UV RESISTANCE LOCUS 8 (UVR8) (Wu et al. 2012; Huang and Deng 2013; Galvao and Fankhauser 2015; Yin and Ulm 2017; Paik and Huq 2019). Among these, phyB serves as the dominant photoreceptor for red light perception, whereas phyA primarily mediates far-red light sensing. Phytochromes have 2 photoconvertible conformations in vivo: the red light–absorbing Pr form and the far-red light–absorbing Pfr form. In the dark, phytochromes are synthesized in Pr form in the cytosol. Upon exposure to visible light, the Pr form of phytochrome is converted to the biologically active Pfr form and translocated into the nucleus, where it interacts with numerous components to transduce the light signaling cascade (Fankhauser and Chen 2008; Cheng et al. 2021).

CONSTITUTIVELY PHOTOMORPHOGENIC1 (COP1) is a central switch that represses photomorphogenesis and acts directly downstream of various photoreceptors. COP1 was first identified in *Arabidopsis thaliana* by genetic screens for dark-grown mutants that mimicked wild-type seedlings grown in the light (von Arnim and Deng 1994). The COP1 protein consists of 3 recognizable domains: an N-terminal RING finger motif, followed by a Coiled-coil domain, and 7 WD40 repeats. Known as a RING E3 ubiquitin ligase, COP1 targets a wealth of substrates for ubiquitination and degradation via the 26S proteasome system (Lau and Deng 2012; Han et al. 2020). SUPPRESSOR OF PHYTOCHROME A (SPA1), containing an N-terminal kinase-like domain, a central Coiled-coil domain, and a C-terminal WD-repeat domain, was first characterized as a repressor of a weak *phyA* mutation (Hoecker et al. 1998, 1999). There are 3 SPA1 homologs, namely SPA2, SPA3, and SPA4. Mutation in each individual SPA protein causes a weak or no phenotype defect, while the quadruple *spa* mutant displays a constitutively photomorphogenic phenotype similar to that of a lethal *cop1* mutant allele (*cop1-5*) (Laubinger and Hoecker 2003; Laubinger et al. 2004; Ishikawa et al. 2006). In the dark, 2 SPA molecules and 2 COP1 molecules form a tetrameric configuration of the heterogeneous COP1–SPA core complex to promote photomorphogenesis-promoting factors, such as ELONGATED HYPOCOTYL 5 (HY5) for ubiquitination and degradation, thus repressing photomorphogenesis in *Arabidopsis* (Osterlund et al. 2000; Zhu et al. 2008; Chen et al. 2010). With visible light irradiation, photoactivated phyA/B and CRY1 interact with SPAs, accelerating the dissociation of COP1-SPA complex, which results in the accumulation of HY5 and facilitating photomorphogenesis (Lian et al. 2011; Liu et al. 2011; Lu et al. 2015; Sheerin et al. 2015). However, light-excited CRY2 interacts with COP1, which attenuates the interaction between COP1 and its substrates, leading to CO accumulation and flowering promotion (Zuo et al. 2011; Ponnu et al. 2019).

PHYTOCHROME-INTERACTING FACTORS (PIFs) are the seventh subfamily of basic helix-loop-helix (bHLH) transcription factors and are highly accumulated in the dark, negatively regulating plant photomorphogenesis (Bailey et al. 2003; Heim et al. 2003). PIF3 was the first member identified to interact with phyB in a yeast two-hybrid screen (Ni et al. 1998). In addition to the bHLH domain, the PIF3 protein carries another 2 motifs, termed the active phytochrome A–binding (APA) and the active phytochrome B–binding (APB) motifs, which are responsible for the interaction with the Pfr forms of phyA and phyB, respectively (Leivar and Quail 2011). Upon light exposure, photoactivated phytochromes translocate from the cytoplasm to the nucleus and interact with PIF3, which promotes the following phosphorylation and degradation through the ubiquitin-mediated 26S proteasome pathway. Recent structural studies revealed that the phyB dimer exhibits slight asymmetry, resulting in the binding of only one PIF6 molecule (Wang et al. 2024). Several groups of protein kinases have been reported to phosphorylate PIF3, including BRASSINOSTEROID-INSENSITIVE 2 (BIN2) (Ling et al. 2017), PHOTOREGULATORY PROTEIN KINASES (PPKs) (Ni et al. 2017), mitogen-activated protein kinase (MAPK) MPK6 (Xin et al. 2018) and phytochromes (Shin et al. 2016). Two groups of E3 ubiquitin ligases have been shown to promote PIF3 degradation. The cullin1-based EIN3-BINDING F-BOX PROTEIN 1/2 (EBF1/2) ubiquitinate phosphorylated PIF3 to induce its degradation under a wide range of light intensities, while the cullin3-based LIGHT RESPONSE BTB 1/2/3 (LRB1/2/3) E3 ubiquitin ligase target phyB and phosphorylated PIF3 for ubiquitination and concurrent degradation under high light conditions (Ni et al. 2014; Dong et al. 2017; Jiang et al. 2017).

BR-ENHANCED EXPRESSION 1 (BEE1), BEE2, and BEE3 are another group of bHLH transcription factors that belong to the twelfth subfamily and positively regulate brassinosteroid (BR) signaling (Friedrichsen et al. 2002; Toledo-Ortiz et al. 2003; Yin et al. 2005). Previous studies have revealed that BEE1/2/3 and BIM1/2/3 positively regulate hypocotyl elongation in response to shade and are required for plant viability (Cifuentes-Esquivel et al. 2013). Besides, BES1 activates the transcription of *BEE1*, the gene products of which directly binds to the *FT* promoter to induce its transcription and promote flowering (Wang et al. 2019). Blue light receptor CRY2 interacts with BEE1, which promotes the binding ability of BEE1 toward *FT* promoters, further stimulating photoperiodic flowering. In addition, photomorphogenic repressors BBX28 and BBX29 associate with BEE1/2/3 to enhance the binding ability and transcriptional activity of BEE1/2/3 to their target genes, facilitating BR-mediated skotomorphogenesis (Lin et al. 2018; Cao et al. 2022; Cao et al. 2023). Although BEE1/2/3 are known to be involved in controlling shade avoidance, flowering, light, and BR signaling, the specific pathways and mechanisms by which BEE1/2/3 mediate these responses have not yet been established.

In this study, we demonstrated that the bHLH-type transcription factors BEE1, BEE2, and BEE3 negatively regulate plant photomorphogenesis. Light promoted the degradation of BEE1/2/3 through the 26S proteasome-dependent pathway. BEE1/2/3 interacted with phyA/B, which interfered with the phyA/B-PIF3 interaction, thereby increasing PIF3 accumulation and repressing photomorphogenesis. Besides, BEE1/2/3 interacted with SPA1/4, which forms a complex with COP1 to stabilize BEE1/2/3. Mutations in *BEE1/2/3* enhanced the constitutive photomorphogenic phenotype of *spa1 spa2 spa4*. Additionally, BEE1/3 significantly attenuated the interaction between phyA/B and SPA1 to further repress the hyperactive light signaling. Altogether, these findings demonstrate that BEE1/2/3 interact with phyA/B to directly transduce the light signal into the nucleus to promote PIF3 accumulation, as well as interact with SPA proteins to interfere with the phyA/B-SPA1 complex, alleviating exaggerated active light signaling and ensuring proper seedling development.

## Results

### BEE1, BEE2, and BEE3 are negative regulators of light signaling

Previous study indicates that BEE1/2/3 act as repressors of light signaling (Cao et al. 2022); however, the underlying molecular mechanism remains unclear. To this end, we first analyzed the phenotypes of *bee1 bee2 bee3* mutant and overexpressing transgenic lines. Under different light conditions, the *bee1 bee2 bee3* triple mutant developed shortened hypocotyls, while *35S:MYC-BEE1* Col (hereafter referred to as *MYC-BEE1* Col) and *35S:YFP-BEE3* Col (hereafter referred to as *YFP-BEE3* Col) transgenic lines exhibited elongated hypocotyls (Figure S1), consistent with previous data showing that BEE1/2/3 act as negative regulators of light signaling (Cao et al. 2022). The comparable hypocotyl lengths observed in *35S:YFP-BEE2* Col seedlings (hereafter referred to as *YFP-BEE2* Col) relative to the wild-type, under various light conditions, could be largely attributed to the modestly elevated expression and protein accumulation of BEE2 (Cao et al. 2022). To determine whether light regulates the transcript and protein levels of *BEE1/2/3*, we conducted quantitative real-time reverse transcriptase PCR (RT-qPCR) and immunoblot assays using Col and transgenic lines overexpressing *BEE1/2/3* at various light conditions (white 92.65 μmol/m^2^/s, blue 4.34 μmol/m^2^/s, red 109.9 μmol/m^2^/s, and far-red 4.41 μmol/m^2^/s). Our results indicated that constant white and blue light slightly reduced the transcription of *BEE1*, while constant white and red light significantly increased the transcription of *BEE2* and *BEE3* (Fig. 1a to 1c). When 4-day-old (4-d-old) etiolated Col seedlings were transferred to white light (92.65 μmol/m^2^/s) for different times ranging from 1 to 96 h, we observed that the endogenous *BEE1/2/3* expression underwent a transient increase before being reduced under prolonged light exposure (Figure S2). Immunoblots showed that darkness promoted the protein accumulation of MYC-BEE1 and YFP-BEE3 which appear to be degraded in white, blue, and far-red light conditions, whereas YFP-BEE2 was unstable and readily degraded across all light conditions (Fig. 1d to 1i). When 4-d-old etiolated *MYC-BEE1* Col seedlings were transferred to white light for periods ranging from 1 to 96 h, MYC-BEE1 levels increased within 48 h of exposure to white light but decreased after prolonged light irradiation (Fig. 1j). Similarly, YFP-BEE3 became degraded when 4-d-old etiolated *YFP-BEE3* Col seedlings were transferred to white light for 48 h (Fig. 1l). However, YFP-BEE2 degraded when 4-d-old etiolated *YFP-BEE2* Col seedlings were transferred to white light for only 2 h (Fig. 1k). The rapid turnover of BEE2 protein also likely resulted in hypocotyl lengths in *YFP-BEE2* Col seedlings that were comparable to those of the wild-type under various light conditions. Moreover, this observation points to a functional divergence of BEE2 from BEE1 and BEE3. Intriguingly, MG132, the inhibitor of the 26S proteasome, significantly suppressed the protein degradation of MYC-BEE1 and YFP-BEE2/3; the increased protein accumulation of BEE1/2/3 became more obvious as the MG132 concentration increased (Fig. 1m to 1o). Thus, BEE1/2/3 underwent 26S proteasome-dependent degradation. Taken together, our data demonstrate that the transcription and protein stability of BEE1/2/3 are tightly regulated by light.

**Figure 1:**
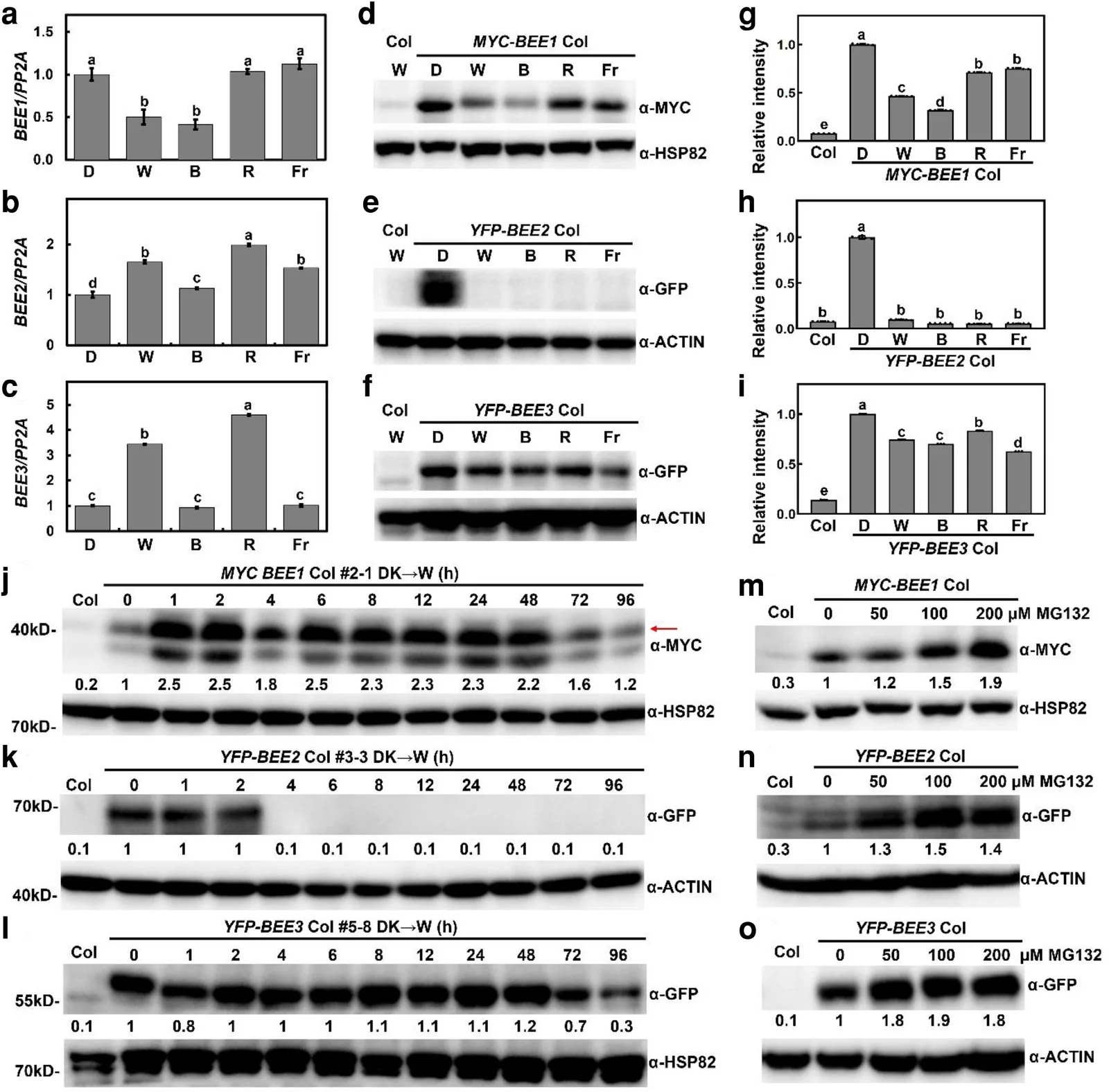
Light regulates BEE1/2/3 gene expression and protein stability. (a–c) *BEE1*, *BEE2*, and *BEE3* transcript levels in 4-d-old Col grown in the dark, white, blue, red, and far-red light (***P < 0.001). Error bars represent SD of 3 technical replicates. (d–f) Immunoblot assay showing MYC-BEE1 (d), YFP-BEE2 (e), and YFP-BEE3 (f) protein levels in 4-d-old Col, *MYC-BEE1* Col, *YFP-BEE2* Col, and *YFP-BEE3* Col grown in the dark, white (92.65 μmol/m^2^/s), blue (4.34 μmol/m^2^/s), red (109.9 μmol/m^2^/s), and far-red (4.41 μmol/m^2^/s) light conditions. MYC-BEE1 proteins were detected with α-c-MYC antibody (Sigma-Aldrich). YFP-BEE2 and YFP-BEE3 proteins were detected with α-GFP antibody (Abmart). HSP82 and ACTIN served as loading controls. (g–i) Relative protein intensity of MYC-BEE1 (g), YFP-BEE2 (h), and YFP-BEE3 (i). (j) Immunoblot assay showing MYC-BEE1 protein levels in 4-d-old *MYC-BEE1* Col grown in the dark and transferred to white light (92.65 μmol/m^2^/s) for the indicated time. Values indicate the relative band density of MYC-BEE1 protein normalized to that of the loading control HSP82 via ImageJ software. (k and l) Immunoblot assay showing YFP-BEE2 (k) and YFP-BEE3 (l) protein levels in 4-d-old *YFP-BEE2* Col and *YFP-BEE3* Col grown in the dark and transferred to white light (92.65 μmol/m^2^/s) for the indicated time. Values indicate the relative band density of YFP-BEE2 protein normalized to that of the loading control ACTIN via ImageJ software. (m) Immunoblot assay showing MYC-BEE1 protein levels in 4-d-old *MYC-BEE1* Col grown in the dark and then transferred to blue light for 12 hours with and without MG132. Values indicate the relative band density of MYC-BEE1 protein normalized to that of the loading control HSP82 via ImageJ software. (n and o) Immunoblot assay showing YFP-BEE2 (n) and YFP-BEE3 (o) protein levels in 4-d-old *YFP-BEE2* Col and *YFP-BEE3* Col grown in the white light with and without MG132. Values indicate the relative band density of YFP-BEE2/3 protein normalized to that of the loading control ACTIN via ImageJ software.

### BEE1/2/3 physically interact with phyA and phyB

To examine the molecular roles of BEE1/2/3 in light signaling, we carried out yeast two-hybrid assay to screen their interacting partners. Two candidate proteins, phyA and phyB, the far-red and red light receptors, physically interacted with BEE1/2/3. In our yeast two-hybrid assays, BEE1, BEE2, and BEE3 interacted with phyA in the pLexA system, while BEE2 and BEE3 interacted with phyB in the Gal4 system (Fig. 2a and 2b). However, BEE1 did not interact with phyB in the Gal4 system, likely because the protein expression level of BEE1-pGBK was too little in the yeast cell, which was found to be similar to the interaction between BBX28 and BEE1 (Cao et al. 2022). Moreover, domain-mapping assays revealed that BEE1/2/3 interacted with the C-termini of phyA (563 to 1,123 amino acids [aa]) but did not associate with the N-terminus of phyA (1 to 617 aa), despite phyA-C1-pLexA fusion protein showing autoactivation (Fig. 2c to 2e). In addition, the N-terminal fragment of phyB (1 to 653 aa) interacted with the full-length BEE1, the N-terminal region of BEE1 (1 to 120 aa), the C-terminal region of BEE1 (120 to 260 aa), the full-length BEE2, the N-terminal region of BEE2 (1 to 120 aa), C-terminal region of BEE2 (120 to 304 aa), the full-length BEE3, and the C-terminal region of BEE3 (118 to 261 aa), while the C-terminal fragment of phyB (600 to 1,172 aa) interacted with the N-terminal region of BEE1 (1 to 120 aa), C-terminal region of BEE1 (120 to 260 aa), the full length of BEE2, C-terminal region of BEE2 (120 to 304 aa), the full length of BEE3, and the C-terminal region of BEE3 (118 to 261 aa) (Fig. 2f; Figure S3). Notably, the BEE2-pGBKT7 fusion protein showed only weak autoactivation after 3 days of incubation on the SD/-Leu-Trp-Ade-His plates. Together, these data suggest that the C-terminus of phyA is required for its interaction with BEE1/2/3, while both the N-termini and C-termini of phyB are responsible for its interaction with BEE1/2/3, especially with the C-termini of BEE1/2/3.

**Figure 2:**
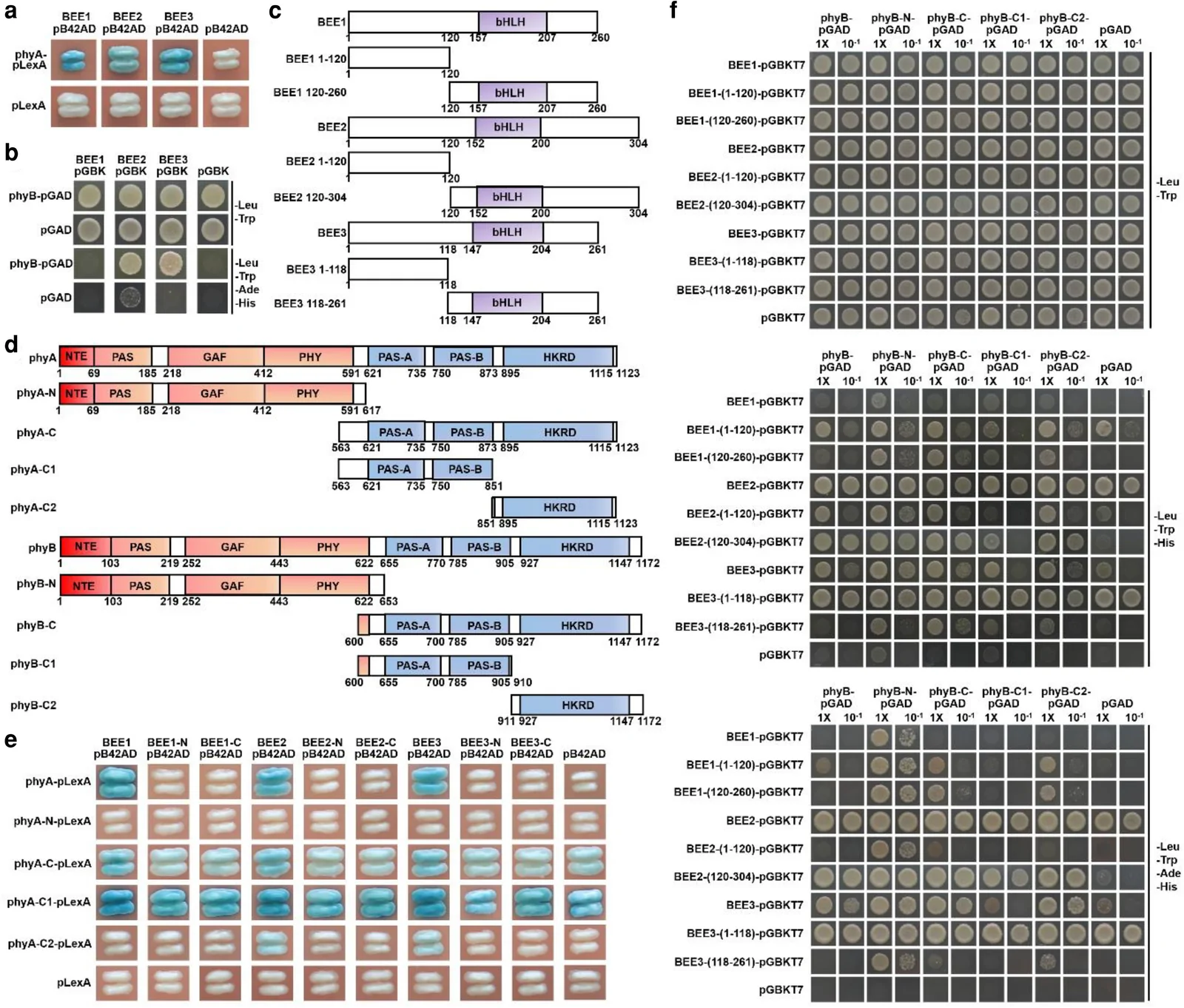
BEE1/2/3 interact with phyA/B in yeast. (a and b) Yeast two-hybrid assay of interactions between the indicated phyA/B and BEE1/2/3 in yeast. Yeast two-hybrid assay of interactions between BEE1/2/3 and phyA in the pLexA system. phyA were introduced into pLexA. BEE1/2/3 were introduced into pB42AD. EGY48 (p8oLacZ) yeast strains containing the indicated vectors were grown on SD/-Trp-His-Ura or SD/Raf/Gal-Trp-His-Ura with X-Gal (a). Yeast two-hybrid assay of interactions between phyB and BEE1/2/3 in the Gal4 system. phyB was fused to GAL4 DAD in pGADT7. BEE1/2/3 were fused to GAL4 DBD in pGBKT7. AH109 yeast strains containing the indicated vectors were grown on either SD/-Leu-Trp or SD/-Leu-Trp-Ade-His (b). (c and d) Diagram of truncated BEE1/2/3 (c) and phyA/B (d). (e) Yeast two-hybrid assay of interactions between the indicated phyA and BEE1/2/3 in the pLexA system. A series of truncation fragments of phyA were introduced into pLexA. The N-terminus and C-terminus of BEE1/2/3 were introduced into pB42AD. EGY48 (p8oLacZ) yeast strains containing the indicated vectors were grown on either SD/-Trp-His-Ura or SD/Raf/Gal-Trp-His-Ura with X-Gal. (f) Yeast two-hybrid assay of interactions between the indicated phyB and BEE1/2/3 in the Gal4 system. A series of truncation fragments of phyB were fused to GAL4 DAD in pGADT7. The N-terminus and C-terminus of BEE1/2/3 were fused to GAL4 DBD in pGBKT7. AH109 yeast strains containing the indicated vectors were grown on either SD/-Leu-Trp for 3 days or SD/-Leu-Trp-His for 3 days or SD/-Leu-Trp-Ade-His for 5 days.

Next, the phyA/B-BEE1/2/3 interactions were confirmed by bimolecular fluorescence complementation (BiFC) assays in planta. Strong YFP signals were observed in *Nicotiana benthamiana* leaf cells transiently co-expressing phyA with the N-terminus of YFP (phyA-YFP^N^) and BEE1/2/3 with the C-terminus of YFP (BEE1/2/3-YFP^C^) (Fig. 3a). However, the negative controls phyA-YFP^N^ and YFP^C^, YFP^N^ and BEE1/2/3-YFP^C^, and phyA-YFP^N^ and ARGONAUTE6 (AGO6)-YFP^C^ did not produce any detectable YFP signals in *N. benthamiana* leaves (Fig. 3a). Strong YFP signals were similarly reconstituted when we transiently co-expressed phyB with the C-terminus of YFP (phyB-YFP^C^) and BEE1/2/3 with the N-terminus of YFP (BEE1/2/3-YFP^N^) in *N. benthamiana* leaf cells, while YFP signals were not observed when we transiently co-expressed the negative controls phyB-YFP^C^ with YFP^N^, and phyB-YFP^C^ with AGO6-YFP^N^ in *N. benthamiana* leaves (Fig. 3a). We then performed co-immunoprecipitation (Co-IP) assays using *N. benthamiana* leaves transiently expressing YFP-phyA and MYC-BEE1/3. As shown in Fig. 3b and 3d, YFP-phyA co-immunoprecipitated with MYC-BEE1/3 which were immunoprecipitated by MYC beads. When YFP-phyA and BEE2-FLAG were transiently expressed in *N. benthamiana* leaves, YFP-phyA co-immunoprecipitated with BEE2-FLAG which was immunoprecipitated by FLAG beads (Fig. 3c). Similarly, FLAG-phyB and YFP-BEE1/2/3 were transiently expressed in *N. benthamiana* leaves, and FLAG-phyB co-immunoprecipitated with YFP-BEE1/2/3 using GFP beads (Fig. 3e to 3g). Collectively, these data provide further evidence of the association between phyA/B and BEE1/2/3 in vivo.

**Figure 3:**
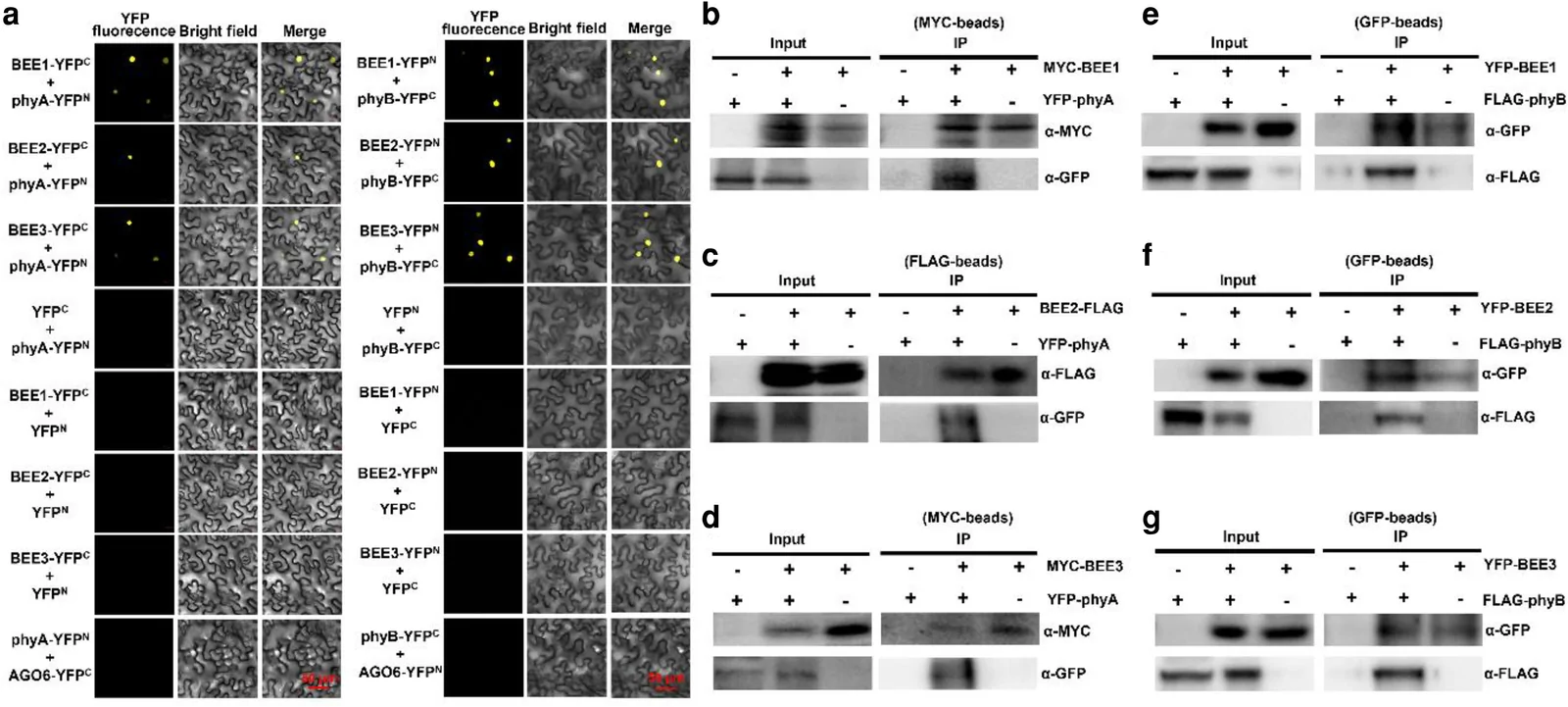
BEE1/2/3 interact with phyA/B in vivo. (a) BiFC assay showing the interaction between phyA/B and BEE1/2/3. Full-length phyA/B and BEE1/2/3 were fused to the split C- or N-terminal (YFP^C^ or YFP^N^) fragments of YFP. Unfused YFP N-terminal (YFP^N^) or YFP C-terminal (YFP^C^) fragments and AGO6-YFP^C^ were used as negative controls. Merge, merged images of YFP channel and brightfield. Bar = 50 μm. (b and d) Co-IP analysis showing that YFP-phyA interacts with MYC-BEE1/3. Total proteins were extracted from *N. benthamiane* leaves transiently expressing 35S:YFP-phyA, 35S:MYC-BEE1/3, or their combination. MYC-BEE1 (b) and MYC-BEE3 (d) proteins were immunoprecipitated with α-MYC beads (Alpalife), and the co-immunoprecipitated YFP-phyA were detected with an α-GFP antibody (Abmart). (c) Co-IP analysis showing that YFP-phyA interacts with BEE2-FLAG. Total protein was extracted from *N. benthamiana* leaves transiently expressing 35S:YFP-phyA, 35S:BEE2-FLAG, or their combination. BEE2-FLAG proteins were immunoprecipitated with α-FLAG beads (Alpalife), and the co-immunoprecipitated YFP-phyA were detected with an α-GFP antibody (Abmart). (e–g) Co-IP analysis showing that FLAG-phyB interacts with YFP-BEE1/2/3. Total proteins were extracted from *N. benthamiana* leaves transiently expressing 35S:YFP-BEE1/2/3, 35S:FLAG-phyB, or their combination. YFP-BEE1/2/3 proteins were immunoprecipitated with α-GFP beads (Alpalife), and the co-immunoprecipitated FLAG-phyB were detected with an α-FLAG antibody (Sigma-Aldrich).

### BEE1/2/3 genetically interact with phyA and phyB

To explore the genetic interactions between BEE1/2/3 and PHYA/B in light signaling, we generated the *phyA phyB bee1 bee2 bee3* quintuple mutant by crossing *phyA phyB* with *bee1 bee2 bee3*. In various wavelength-specific light conditions (W, B, R, and FR), compared to the wild-type, the *phyA phyB* double mutant developed longer hypocotyls, while the *bee1 bee2 bee3* triple mutant exhibited shorter hypocotyls. However, the hypocotyl lengths of the *phyA phyB bee1 bee2 bee3* quintuple mutant were shorter than that of the *phyA phyB* double mutant but longer than that of the *bee1 bee2 bee3* triple mutant under various light conditions tested (W, B, R, and FR) (Fig. 4a to 4h). Therefore, these results demonstrate that *BEE1/2/3* partially act downstream of *PHYA/B* to regulate hypocotyl elongation.

**Figure 4:**
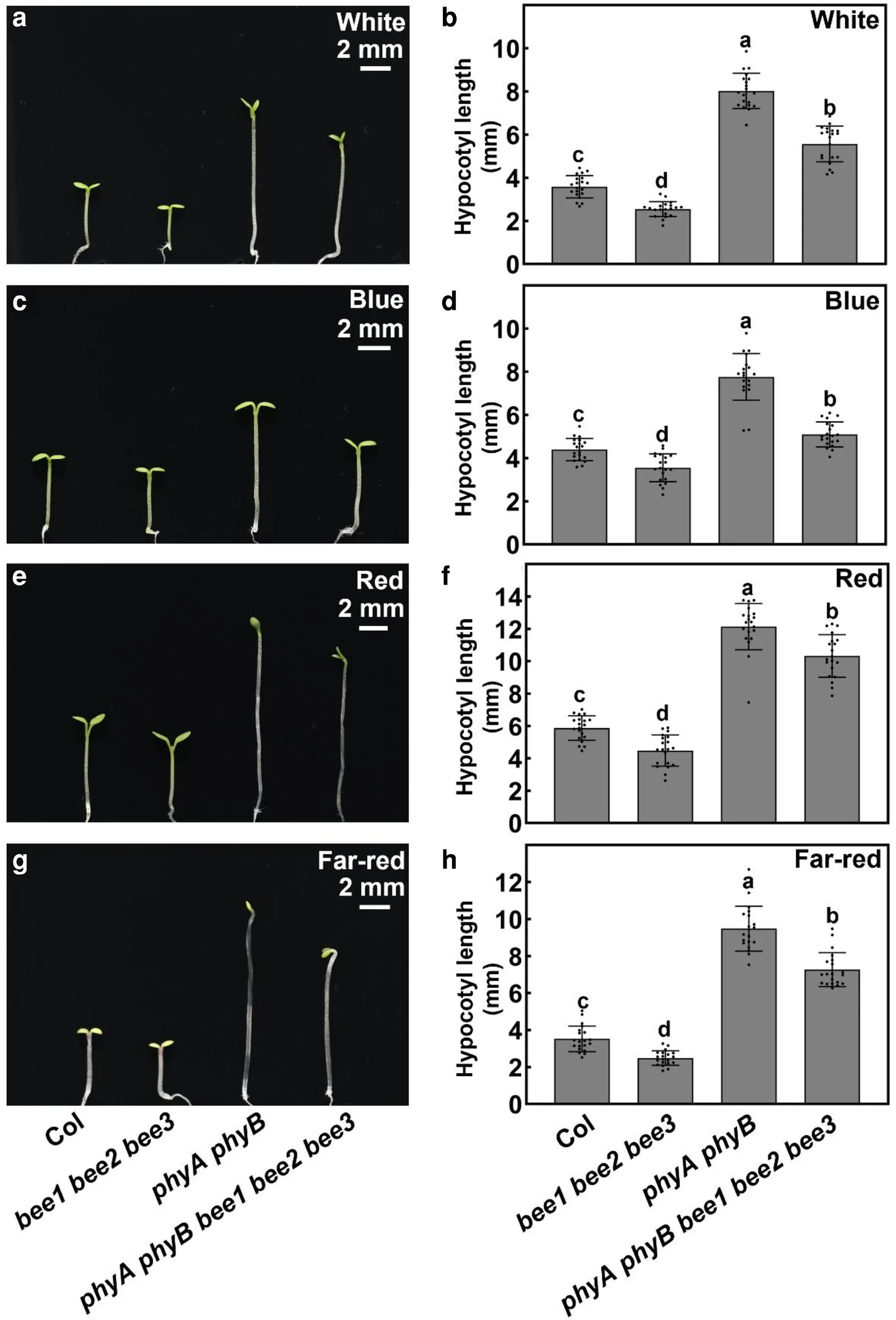
*BEE1/2/3* partially act downstream of *PHYA/B*. (a–h) Hypocotyl lengths and phenotypes of 4-d-old Col, *bee1 bee2 bee3*, *phyA phyB*, and *phyA phyB bee1 bee2 bee3* seedlings grown in W (10.35 μmol/m^2^/s) (a and b), B (5.21 μmol/m^2^/s) (c and d), R (86.8 μmol/m^2^/s) (e and f), and FR (7.98 μmol/m^2^/s) (g and h) light conditions. Hypocotyl length is expressed in mm. Data are means ± SE; n ≥ 20. Different letters above the bars indicate significant differences (*P* < 0.05), as determined by one-way ANOVA with Tukey's post hoc analysis. The experiments were performed 3 times with similar results. The graphs depict 1 of 3 experiments.

Based on the observation that overexpression of *BEE1* and *BEE3*, like overexpressing *PIF3*, resulted in dramatic elongated hypocotyls, we thus used these transgenic plants for further analysis. We then crossed *MYC-BEE1* Col and *YFP-BEE*3 Col overexpression transgenic lines with *phyA phyB* double mutant to generate *MYC-BEE1 phyA phyB* and *YFP-BEE3 phyA phyB*. In various light conditions, both the *phyA phyB* double mutant and *BEE1/3* overexpression transgenic lines exhibited longer hypocotyls than the wild-type, while the *phyA phyB* double mutant had longer hypocotyls than *MYC-BEE1* Col and *YFP-BEE3* Col transgenic seedlings. However, the hypocotyl lengths of *MYC-BEE1 phyA phyB* were longer than that of *MYC-BEE1* Col but shorter than that of the *phyA phyB* double mutant in the white, red, and far-red light (Figure S4). Similarly, the hypocotyl lengths of *YFP-BEE3 phyA phyB* were longer than that of *YFP-BEE3* Col but shorter than that of the *phyA phyB* double mutant in the white, red, and far-red light (Figure S4). Together, these results suggest that *BEE1/2/3* are partially epistatic to *phyA/phyB* with respect to hypocotyl elongation.

### BEE1/2/3 promote PIF3 protein stability

phyA and phyB have been reported to interact with PIF3 to accelerate its phosphorylation and degradation (Ni et al. 1998). To test whether phyA/B regulated the stability of BEE1 and BEE3, we conducted immunoblot assays to compare the protein levels of BEE1 and BEE3 in Col and the *phyA phyB* double mutant grown under white and red light. The protein abundance of BEE3 in Col were less than those in the *phyA phyB* double mutant, indicating that phyA and phyB negatively regulate the protein stability of BEE3 in the white and red light (Figure S5). We then determined whether BEE1/3 modulated the interactions between phyA/B and PIF3. Pull-down assays were performed using MBP-PIF3, GST-BEE1, His-Trigger Factor-phyA (His-TF-phyA), and His-phyB-N proteins, which were expressed and purified from *Escherichia coli*. Given the challenge of expressing and purifying full-length phyB in pET28a, we turned to the His-phyB-N construct as a viable alternative. Our results demonstrated that GST-BEE1 and GST-BEE3 each interacted with His-TF-phyA and His-phyB-N, while also simultaneously and markedly weakening the phyA/B-PIF3 interaction (Fig. 5a to 5d). Moreover, a direct interaction was observed between MBP-PIF3 and GST-BEE1/3. Furthermore, we performed semi-Co-IP assays by transiently expressing FLAG-phyB and MYC-PIF3 in tobacco leaves. Our immunoblot data indicated that FLAG-phyB were able to co-immunoprecipitate with MYC-PIF3; however, the addition of GST-BEE1 or GST-BEE3 notably alleviated the association between phyB and PIF3 (Fig. 5e).

**Figure 5:**
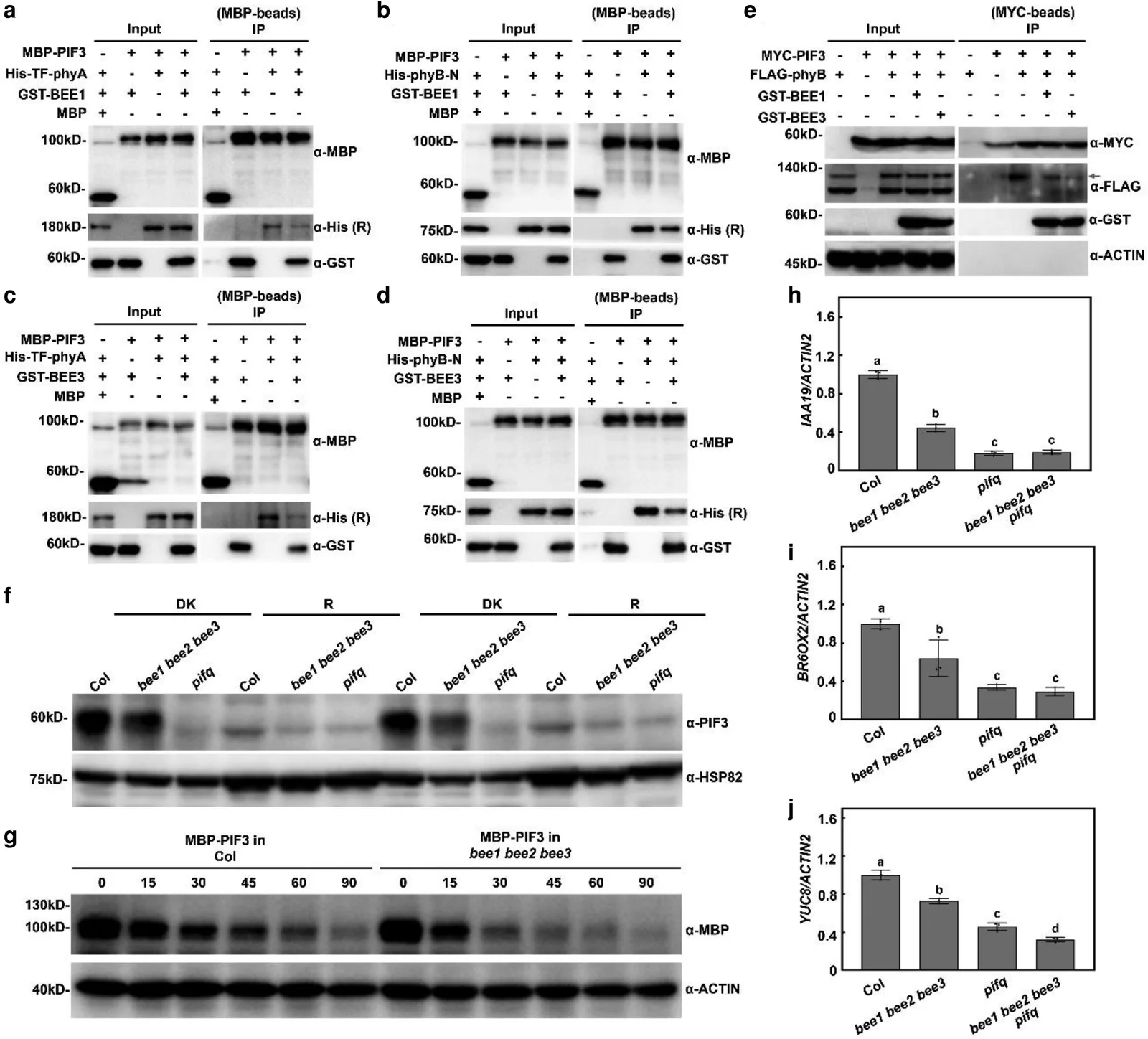
BEE1/2/3 promote PIF3 protein accumulation. (a) Pull-down assay showing that BEE1 inhibits the interaction between phyA and PIF3. The indicated combinations of His-TF-phyA, MBP-PIF3, MBP, and GST-BEE1 were purified in *E. coli*. The precipitated proteins were analyzed by immunoblotting with antibodies against His (CMCTAG), MBP (CMCTAG), and GST (CMCTAG), respectively. (b) Pull-down assay showing that BEE1 inhibits the interaction between phyB and PIF3. The indicated combinations of His-phyB-N, MBP-PIF3, MBP, and GST-BEE1 were purified in *E. coli*. The precipitated proteins were analyzed by immunoblotting with antibodies against His, MBP, and GST, respectively. (c) Pull-down assay showing that BEE3 inhibits the interaction between phyA and PIF3. The indicated combinations of His-TF-phyA, MBP-PIF3, MBP, and GST-BEE3 were purified in *E. coli*. The precipitated proteins were analyzed by immunoblotting with antibodies against His, MBP, and GST, respectively. (d) Pull-down assay showing that BEE3 inhibits the interaction between phyB and PIF3. The indicated combinations of His-phyB-N, MBP-PIF3, MBP, and GST-BEE3 were purified in *E. coli*. The precipitated proteins were analyzed by immunoblotting with antibodies against His, MBP, and GST, respectively. (e) Semi-Co-IP assay showing that BEE1 and BEE3 alleviate the interaction between phyB and PIF3. MYC-PIF3 and FLAG-phyB were expressed in tobacco leaves. GST-BEE1 and GST-BEE3 were purified in *E. coli*. The precipitated proteins were analyzed by immunoblotting with antibodies against MYC (Sigma-Aldrich), FLAG (Sigma-Aldrich), and GST (CMCTAG), respectively. (f) PIF3 protein levels in 4-d-old Col, *bee1 bee2 bee3*, and *pifq* seedlings grown in the dark and red light (109.9 μmol/m^2^/s). Endogenous PIF3 protein levels were assessed using a PIF3-specific antibody, HSP82 served as a loading control. (g) Degradation of recombinant MBP-PIF3 in the cell-free system. Protein extracts were prepared from 4-d-old Col and *bee1 bee2 bee3* seedling in the red light (109.9 μmol/m^2^/s) and then incubated with MBP-PIF3 over the indicated time course. ACTIN was used as a nondegraded loading control. (h–j) *IAA19* (h), *BR6OX2* (i), and *YUC8* (j) transcript levels in 4-d-old Col, *bee1 bee2 bee3,* and *pifq* grown in the red light (109.9 μmol/m^2^/s). Data are means ± SE of 3 technical repeats. Asterisks represent statistically significant differences (***P < 0.001, *P < 0.05), as determined by one-way ANOVA with Tukey's post hoc analysis.

We next investigated whether BEE1/2/3 controlled the transcript and protein level of PIF3 in *Arabidopsis*. To this end, Col, *bee1 bee2 bee3* triple mutant, and *pif* quadruple mutant seedlings were grown in the dark and red light for 4 days. Endogenous transcript and protein level of PIF3 were examined by quantitative real-time PCR and immunoblotting, respectively. While *PIF3* mRNA levels were comparable across genotypes under both conditions (Figure S6), the abundance levels of PIF3 proteins were much lower in the *bee1 bee2 bee3* triple mutant than in the wild-type when grown in the dark for 4 days. However, the levels of PIF3 proteins were degraded dramatically in all genotypes when grown in the red light (Fig. 5f). We then performed cell-free degradation assays to validate the effect of BEE1/2/3 on PIF3. The recombination of MBP-PIF3 degraded obviously faster when incubated with protein extracts from *bee1 bee2 bee3* than those from Col (Fig. 5g). Together, these results suggest that BEE1/2/3 positively controls PIF3 abundance in Arabidopsis.

Given the established role of PIFs in positively regulating the transcription of *IAA19*, *YUC8*, and *BR6OX2* (Zhang et al. 2013), we analyzed the expression of these target genes in the *bee1 bee2 bee3* triple mutant. In agreement, the transcript levels of *IAA19*, *YUC8*, and *BR6OX2* were significantly decreased in the *bee1 bee2 bee3* triple mutant and *pif* quadruple mutant when grown in the red light (Fig. 5h to 5j). These results suggest that BEE1/2/3 interact with phyA/B to attenuate the association between phyA/B and PIF3, contributing to promotion of PIF3 accumulation and its target gene expression.

To further dissect the genetic interplay between *BEE1/2/3* and *PIFs*, we generated the *bee1 bee2 bee3 pifq* septuple mutant by crossing *bee1 bee2 bee3* with *pifq.* Phenotypic analysis of Col, *bee1 bee2 bee3*, *pifq*, and *bee1 bee2 bee3 pifq* was performed under darkness and various light conditions (W, B, R, and FR). Both *bee1 bee2 bee3* and *pifq* developed shorter hypocotyls than the wild-type, with *pifq* exhibiting a more pronounced reduction compared to *bee1 bee2 bee3* triple mutants in the light. The *bee1 bee2 bee3 pifq* septuple mutant seedlings resembled *pifq* in darkness, but they were slightly shorter than *pifq* under all light conditions tested (Figure S7). These results indicate that *PIF* genes are epistatic to *BEE1/2/3* in darkness, whereas they act synergistically and redundantly with *BEE1/2/3* to promote hypocotyl elongation in the light.

### BEE1/2/3 interact with SPA proteins

In the process of screening the interacting partners of BEE1/2/3, we found that SPA proteins also interacted with BEE1/2/3. In our yeast two-hybrid assays, both SPA1 and SPA4 interacted with BEE1/2/3 (Fig. 6a). Moreover, domain-mapping assays revealed that the Coiled-coil domain of SPA1 (amino acids 546 to 696) interacted with the full length and the C-terminus of BEE1 (120 to 260 aa), BEE2 (120 to 304 aa), and BEE3 (118 to 261 aa), but did not associate with the N-termini of BEE1/2 (BEE1/2 1 to 120 aa) or BEE3 (BEE3 1 to 118 aa) (Figure S5B). In addition, the Coiled-coil domain of SPA4 (272 to 465 aa) and WD40 domain of SPA4 (466 to 794 aa), but not the N-terminal truncated fragment of SPA4 (1 to 271 aa), strongly interacted with the full-length and C-terminal region of BEE1/2/3 (Figure S8B). Interestingly, the N-terminus of BEE2 and BEE3 also weakly interacted with SPA4 (Figure S8B). Together, these data suggest that both the Coiled-coil domain of SPA1 and the C-termini of BEE1/2/3 are responsible for their protein–protein interactions, while the Coiled-coil and WD40 domains of SPA4 are required for its interactions with BEE1/2/3.

**Figure 6:**
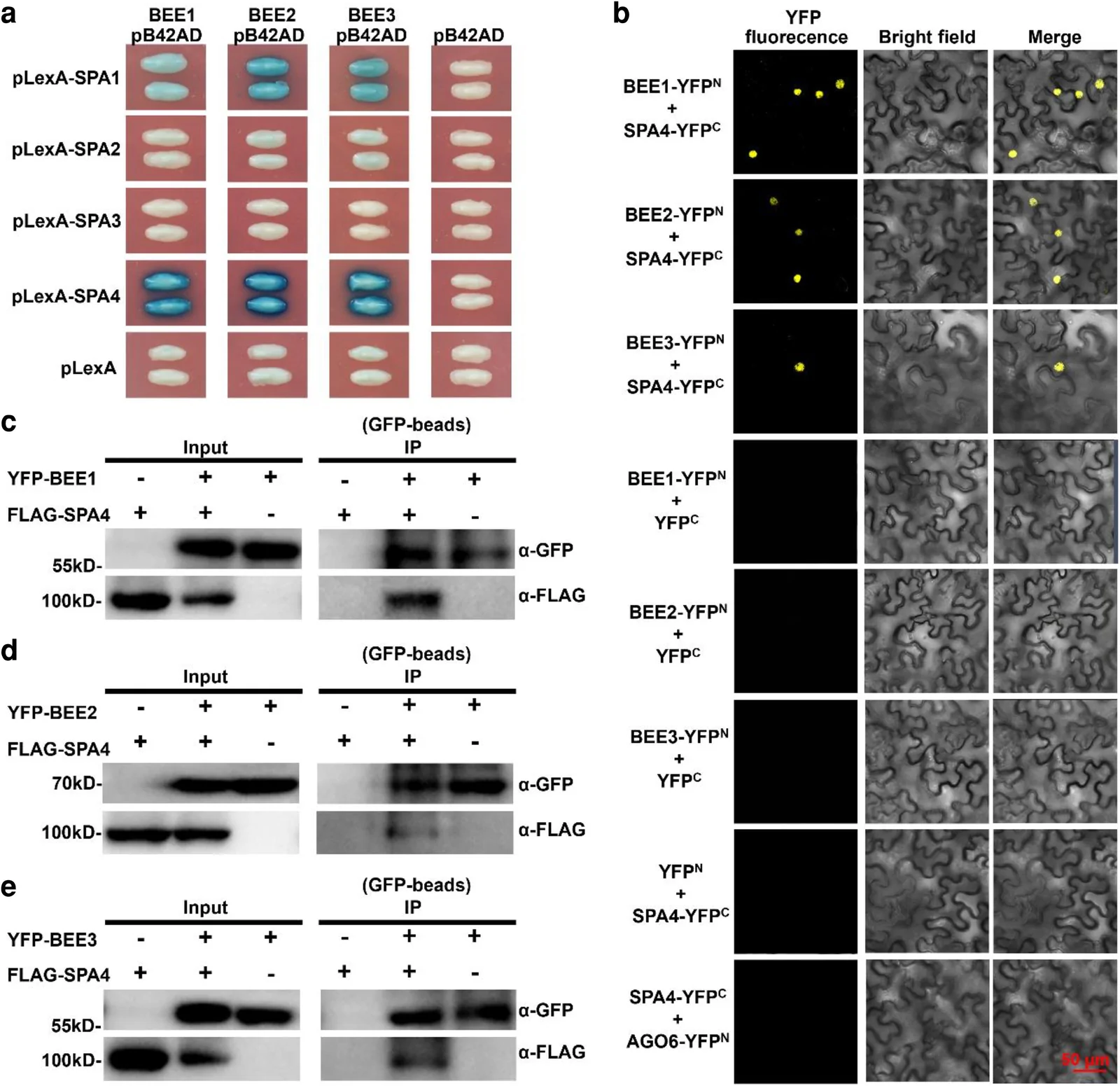
BEE1/2/3 interact with SPA1 and SPA4. (a) Yeast two-hybrid assay of interactions between the indicated BEE1/2/3 and SPA1/4 in the pLexA system. BEE1/2/3 were introduced into pB42AD. SPA1/2/3/4 were introduced into pLexA. EGY48 (p8oLacZ) yeast strains containing the indicated vectors were grown on either SD/-Trp-His-Ura or SD/Raf/Gal-Trp-His-Ura with X-Gal. (b) BiFC assay showing the interaction between BEE1/2/3 and SPA4. Full-length BEE1/2/3 and SPA4 were fused to the split N- or C-terminal (YFP^N^ or YFP^C^) fragments of YFP. Unfused YFP N-terminal (YFP^N^) or YFP C-terminal (YFP^C^) fragments and AGO6-YFP^N^ were used as negative controls. Merge, merged images of YFP channel and bright field. Bar = 50 μm. (c–e) Co-IP analysis showing that FLAG-SPA4 interacts with YFP-BEE1/2/3. Total proteins were extracted from *N. benthamiana* leaves transiently expressing 35S:YFP-BEE1/2/3 and 35S:FLAG-SPA4 together or alone. YFP-BEE1 (c), YFP-BEE2 (d), and YFP-BEE3 (e) proteins were immunoprecipitated with α-GFP beads (Alpalife), and the co-immunoprecipitated FLAG-SPA4 were detected with an α-FLAG antibody (Sigma-Aldrich).

To validate the interactions between SPA1/4 and BEE1/2/3, we conducted bimolecular fluorescence complementation (BiFC) and co-immunoprecipitation (Co-IP) assays. Reconstituted YFP fluorescence were detected in *N. benthamiana* leaf cells transiently co-expressing SPA1/4 with the C-terminus of YFP (SPA1/4-YFP^C^) and BEE1/2/3 with the N-terminus of YFP (BEE1/2/3-YFP^N^) (Fig. 6b; Figure S7C). However, the negative controls SPA1/4-YFP^C^ and YFP^N^, YFP^C^ and BEE1/2/3-YFP^N^, and SPA1/4-YFP^C^ and ARGONAUTE 6 (AGO6)-YFP^N^ did not produce any detectable YFP signals in *N. benthamiana* leaves (Fig. 6b; Figure S7C). We then performed Co-IP assays using *N. benthamiana* leaves transiently expressing FLAG-SPA4 and YFP-BEE1/2/3. As shown in Fig. 6c, 6d, and 6e, FLAG-SPA4 co-immunoprecipitated with YFP-BEE1/2/3. Similarly, Co-IP assay also confirmed that SPA1 interacts with BEE1/2/3 in vivo (Figure S8D to S8E). Altogether, these data provide evidence that both SPA1 and SPA4 physically associate with BEE1/2/3 within plant cells.

### Genetic interaction between BEE1/2/3 and COP1–SPAs

To examine the genetic links between BEE1/2/3 and SPA proteins in light signaling, we generated the *bee1 bee2 bee3 spa1 spa2 spa4* sextuple mutant by crossing *bee1 bee2 bee3* triple mutant with *spa1 spa2 spa4* triple mutant. In various wavelength-specific light conditions (W, B, R, and FR), the *bee1 bee2 bee3* triple mutant and *spa1 spa2 spa4* triple mutant developed shorter hypocotyls than the wild-type, while the *spa1 spa2 spa4* triple mutant had shorter hypocotyls than the *bee1 bee2 bee3* triple mutant (Fig. 7a to 7h). However, the hypocotyl lengths of the *bee1 bee2 bee3 spa1 spa2 spa4* sextuple mutant were comparable with that of *spa1 spa2 spa4* triple mutant in the far-red light at the intensity of 0.95 μmol/m^2^/s (Fig. 7g and 7h) but shorter than that of the *spa1 spa2 spa4* triple mutant under white, blue, and red light conditions (Fig. 7a to 7f), likely due to the potential redundancy within the SPA family.

**Figure 7:**
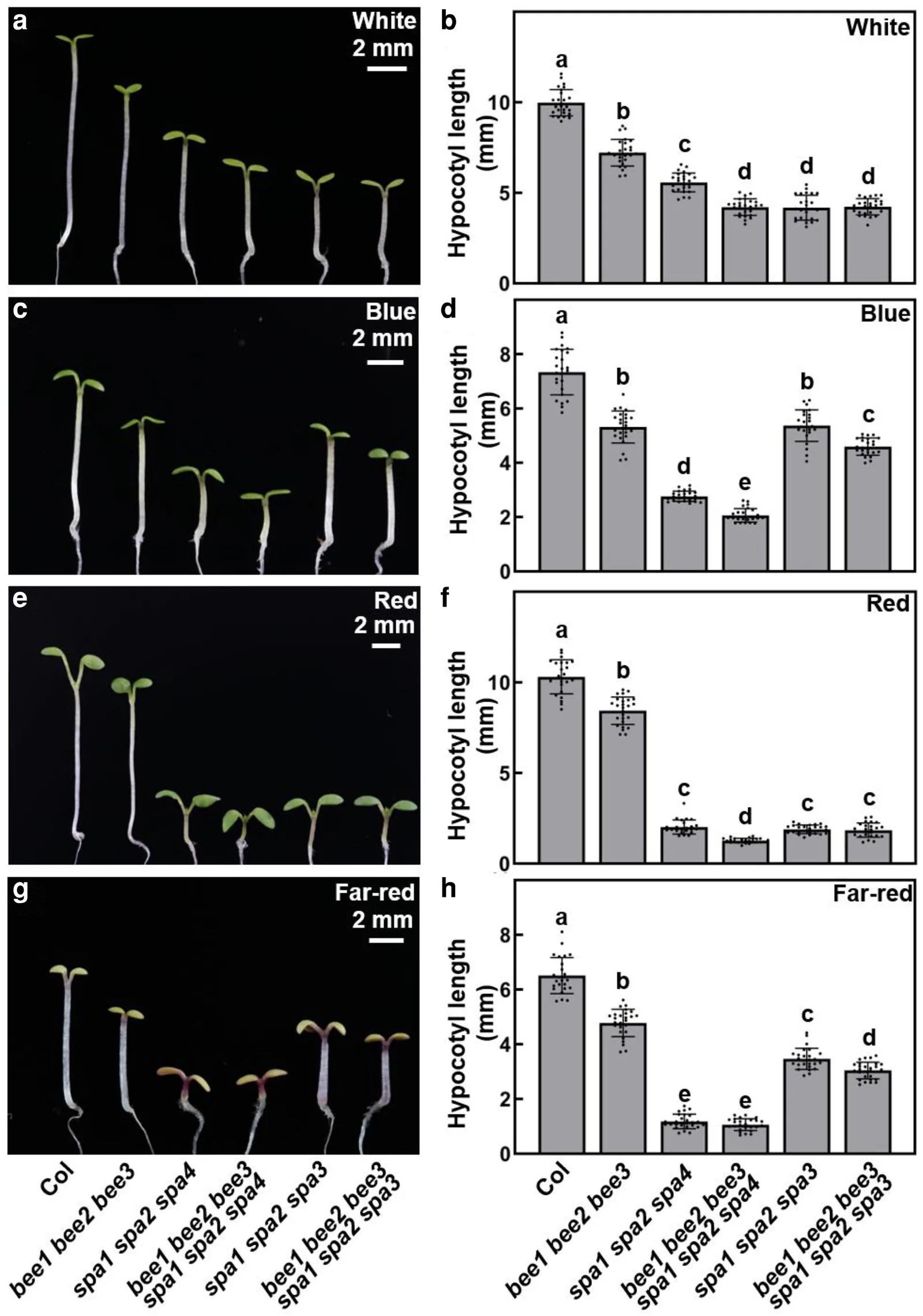
Mutations in *BEE1/2/3* enhance the constitutively photomorphogenic phenotype of *spa1 spa2 spa4*. (a–h) Hypocotyl lengths and phenotypes of 4-d-old Col, *bee1 bee2 bee3*, *spa1 spa2 spa4*, *bee1 bee2 bee3 spa1 spa2 spa4*, *spa1 spa2 spa3*, and *bee1 bee2 bee3 spa1 spa2 spa3* seedlings grown in W (1.7 μmol/m^2^/s) (a, b), B (1.06 μmol/m^2^/s) (c, d), R (15.21 μmol/m^2^/s) (e, f), and FR (0.95 μmol/m^2^/s) (g, h) light conditions. Hypocotyl length is expressed in mm. Data are means ± SE; n ≥ 20. Different letters above the bars indicate significant differences (*P* < 0.05), as determined by one-way ANOVA with Tukey's post hoc analysis. The experiments were performed 3 times with similar results. The graphs depict 1 of 3 experiments.

To further investigate the genetic relationship between *BEE1/2/3* and *SPA1/2/4* under far-red light, we analyzed the phenotypes of Col, *bee1 bee2 bee3*, *spa1 spa2 spa4*, and *bee1 bee2 bee3 spa1 spa2 spa4* seedlings under far-red light at 3 low intensities (0.52 μmol/m^2^/s, 0.317 μmol/m^2^/s, 0.138 μmol/m^2^/s). As shown in Figure S9, the hypocotyl lengths of the *spa1 spa2 spa4* triple mutant and the *bee1 bee2 bee3 spa1 spa2 spa4* sextuple mutant were comparable at 0.52 μmol/m^2^/s and 0.317 μmol/m^2^/s. However, under a very low intensity of 0.138 μmol/m^2^/s, the sextuple mutant exhibited shorter hypocotyls than the *spa1 spa2 spa4* triple mutant. These results suggest that *SPA1/2/4* function partially downstream of *BEE1/2/3* at a low far-red light intensity, but the 2 genetic modules act synergistically under very low far-red light conditions. Together, the genetic interaction between BEE1/2/3 and SPA1/2/4 depends on the far-red light intensity. Therefore, BEE1/2/3 and SPA1/2/4 coordinately promote hypocotyl elongation across white, blue, and red light. More specifically, under far-red light with high intensity, *SPA1/2/4* function partially downstream of *BEE1/2/3* to mediate this process.

In addition, *MYC-BEE1* Col and *YFP-BEE3* Col overexpression transgenic lines were also introduced into the *spa1 spa2 spa4* triple mutant by crossing. In various light conditions, the *spa1 spa2 spa4* triple mutant developed short hypocotyls, while *MYC-BEE1 spa1 spa2 spa4* and *YFP-BEE3 spa1 spa2 spa4* seedlings had longer hypocotyls than *spa1 spa2 spa4* (Fig. 8a to 8d; Figure S10A to S10F). Thus, overexpression of *BEE1* and *BEE3* partially rescued the short hypocotyls of *spa1 spa2 spa4* under dark and light conditions. Moreover, *MYC-BEE1* Col and *YFP-BEE3* Col overexpression transgenic lines were introduced into the *cop1-6* mutant, and we analyzed the phenotypes of *MYC-BEE1 cop1-6* and *YFP-BEE3 cop1-6* plants under various light conditions. As shown in Fig. 8, overexpression of *BEE1* and *BEE3* partially suppressed the short hypocotyls of *cop1-6* (Fig. 8e to 8h; Figure S11A to S11F). Together, these results suggest that *BEE1/2/3* also partially act downstream of COP1 and SPA proteins, and the promotion of hypocotyl elongation by BEE1 and BEE3 is partially dependent on the COP1 and SPA proteins.

**Figure 8:**
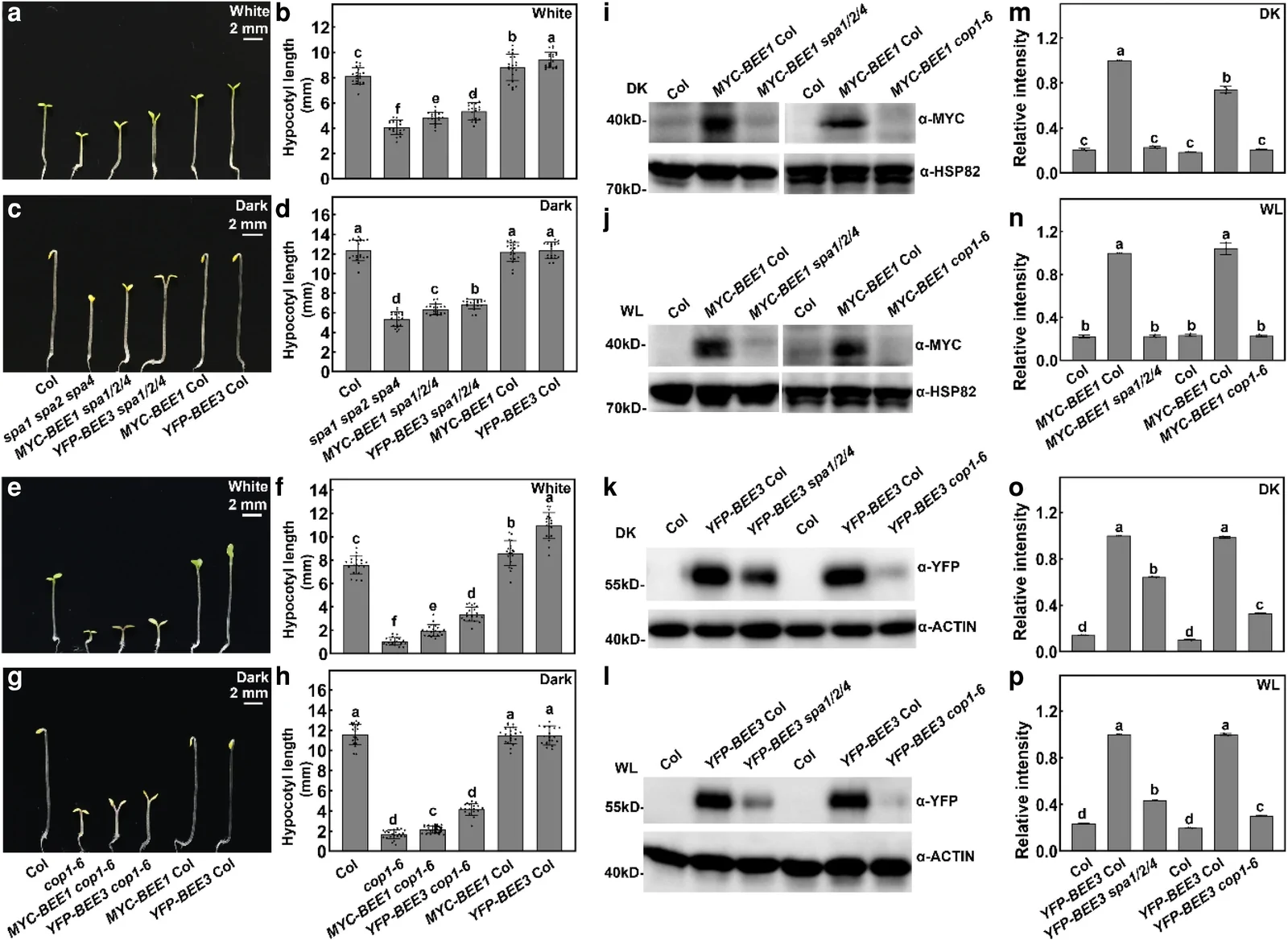
COP1-SPAs complex promotes the protein accumulation of BEE1 and BEE3. (a–d) Hypocotyl lengths and phenotypes of 4-d-old Col, *spa1 spa2 spa4*, *MYC-BEE1 spa1 spa2 spa4*, *YFP-BEE3 spa1 spa2 spa4*, *MYC-BEE1* Col, and *YFP-BEE3* Col seedlings grown in the white light (1.93 μmol/m^2^/s) (a, b) and dark (c, d) conditions. (e–h) Hypocotyl lengths and phenotypes of 4-d-old Col, *cop1-6*, *MYC-BEE1 cop1-6*, *YFP-BEE3 cop1-6*, *MYC-BEE1* Col, and *YFP-BEE3* Col seedlings grown in the white light (1.93 μmol/m^2^/s) (e, f) and dark (g, h) conditions. Hypocotyl length is expressed in mm. *spa1/2/4* indicates the *spa1 spa2 spa4* triple mutant. Data are means ± SE; n ≥ 20. Different letters above the bars indicate significant differences (*P* < 0.05), as determined by one-way ANOVA with Tukey's post hoc analysis. The experiments were performed 3 times with similar results. The graphs depict 1 of 3 experiments. (i and j) Immunoblot assay showing MYC-BEE1 protein levels in 4-d-old Col, *MYC-BEE1* Col, *MYC-BEE1 spa1 spa2 spa*4, and *MYC-BEE1 cop1-6* grown in the dark (i) and white light (j). MYC-BEE1 proteins were detected with α-c-MYC antibody (Sigma-Aldrich). HSP82 served as a loading control. (k and l) Immunoblot assay showing YFP-BEE3 protein levels in 4-d-old Col, *YFP-BEE3* Col, *YFP-BEE3 spa1 spa2 spa4*, and *YFP-BEE3 cop1-6* grown in the dark (k) and white light (l). YFP-BEE3 proteins were detected with α-GFP antibody (Abmart). ACTIN served as a loading control. (m–n) Relative protein intensity of MYC-BEE1 in the dark (m) and white light (n). (o–p) Relative protein intensity of YFP-BEE3 in the dark (o) and white light (p).

Given the interconnection among phyA/B, BEE1/2/3, and SPA1/2/4, we performed transcriptomic analysis of Col (wild-type), *phyA phyB*, *bee1 bee2 bee3*, and *spa1 spa2 spa4* seedlings under red light (120 μmol/m^2^/s). We identified 4,267 genes that displayed statistically significant two-fold (SSTF) changes in the *phyA phyB* mutant (wild-type vs *phyA phyB*, hereafter referred to as phyA/B-regulated genes), 965 genes that displayed SSTF changes in the *bee1 bee2 bee3* mutant (wild-type vs *bee1 bee2 bee3*, hereafter referred to as BEE1/2/3-regulated genes), and 1,955 SSTF genes in the *spa1 spa2 spa4* mutant (wild-type vs *spa1 spa2 spa4*, hereafter referred to as SPA1/2/4-regulated genes). By comparing the expression profiles of these 3 mutants, we found that 434 genes were co-regulated by phyA/B, BEE1/2/3, and SPA1/2/4 (Figure S12A). Among these genes, 74.2% of BEE1/2/3-regulated genes were also regulated by phyA/B, and 57.2% of BEE1/2/3-regulated genes were also regulated by SPA1/2/4 (Figure S12A). We designated these 434 co-regulated genes as members of the phyA/B–BEE1/2/3–SPA1/2/4 pathway and divided them into 2 groups: genes repressed by phyA/B but activated by BEE1/2/3 and SPA1/2/4 (13 genes), and genes activated by phyA/B but repressed by BEE1/2/3 and SPA1/2/4 (2 genes) (Figure S12B to S12D). We then performed gene ontology (GO) analysis and functional clustering of the phyA/B-, BEE1/2/3-, and SPA1/2/4-regulated genes. This analysis revealed that phyA/B-, BEE1/2/3-, and SPA1/2/4-regulated genes were preferentially associated with pathways related to hypoxia, light, cell proliferation, oxidative stress, auxin, jasmonic acid, and other phytohormones (Figure S12G to S12I). These data corroborate the findings from genetic analysis, indicating that BEE1/2/3 genetically interact with both phyA/B and SPA1/2/4 to regulate target gene expression, thereby controlling hypocotyl elongation.

### COP1–SPAs complex promotes BEE1/2/3 accumulation

In light of the genetic and physical interactions between SPA proteins and BEE1/2/3, we assessed the functional interplay between these 2 groups of proteins. BEE1/2/3 are bHLH-type transcription factors, while the COP1–SPAs complex is a RING-type E3 ubiquitin ligase. We considered whether COP1–SPA complex regulates the stability of BEE1/2/3. Accordingly, we evaluated the abundance of BEE1 in Col, *spa1 spa2 spa4*, and *cop1-6* mutant seedlings grown under continuous dark and white light for 4 d. Immunoblot assays showed that the protein accumulation of BEE1 was strongly decreased in the *cop1-6* and *spa1 spa2 spa4* mutants (Fig. 8i and 8j). A pronounced reduction in BEE3 protein abundance was likewise observed in *cop1-6* and *spa1 spa2 spa4* mutants across dark and white light conditions (Fig. 8k and 8l). Interestingly, the protein levels of BEE3 were much lower in the *cop1-6* mutant than in the *spa1 spa2 spa4* triple mutant, likely due to the redundance of SPA proteins. In addition, we conducted cell-free degradation assays using recombinant GST-BEE1/3 incubated with protein extracts from Col and the *cop1-6* and *spa1 spa2 spa4* mutants. The results revealed that the protein amounts of GST-BEE1 and GST-BEE3 dropped more rapidly in *spa1 spa2 spa4* than in Col (Figure S13A and S13B). Similarly, the protein abundance of GST-BEE1 and GST-BEE3 degraded faster in *cop1-6* than in Col (Figure S13C and S13D). Together, these results suggest that the COP1–SPAs complex protects BEE1/3 from degradation under dark and light conditions.

It is well established that photoactivated phyA/B interact with SPAs to interfere with the COP1–SPAs complex, resulting in promoting photomorphogenesis (Lu et al. 2015; Sheerin et al. 2015). Besides, BEE1/2/3 have been found to interact with both phyA/B and SPAs in this study. Therefore, we sought to determine whether BEE1/2/3 play a role in modulating the phyA/B–SPAs interactions. Using modified yeast three-hybrid assays, we found that co-expression of BEE1 or BEE3 notably inhibited the interaction between phyA and SPA1, and between phyB-N and SPA1 (Fig. 9a and 9b). To further verify that BEE1/3 regulated the interaction between phyA/B and SPA1, we performed pull-down assays using recombinant GST-BEE1, GST-BEE3, MBP-SPA1, and His-phyB-N. Our results showed that GST-BEE1 and GST-BEE3 obviously weaken the interaction between phyB-N and SPA1 (Fig. 9c). Using a semi-Co-IP assay in *Nicotiana benthamiana* leaves transiently expressing YFP-phyB and FLAG-SPA1, we assessed the effect of adding GST-BEE1 or GST-BEE3. The immunoblot results indicated that both BEE1 and BEE3 significantly decreased the phyB-SPA1 interaction (Fig. 9d). In addition, *MYC-SPA1* Col was crossed with *phyB-CFP* Col (*PBC*) to generate the homozygous *MYC-SPA1 PBC* transgenic lines that overexpress both *MYC-SPA1* and *phyB-CFP*. *PBC* and *MYC-SPA1 PBC* seedlings were grown under continuous white light for 4 days at 2 intensities: 92.6 μmol/m^2^/s (high light) and 40 μmol/m^2^/s (low light). We then carried out semi-Co-IP assay to assess the phyB-SPA1 interaction in the presence or absence of BEE1/3. The immunoblot results revealed that BEE1 and BEE3 significantly attenuated the interaction between phyB and SPA1 at both low and high light irradiation. Notably, this inhibitory effect was more pronounced under high light than under low light. These results suggest that BEE1 and BEE3 potently alleviate the interaction between phyB and SPA1, particularly under high light intensity (Fig. 9e and 9f). Collectively, our data demonstrate that BEE1 and BEE3 likely disturb the formation of the phyA/B–SPA complex to attenuate plant photomorphogenesis under high light condition.

**Figure 9:**
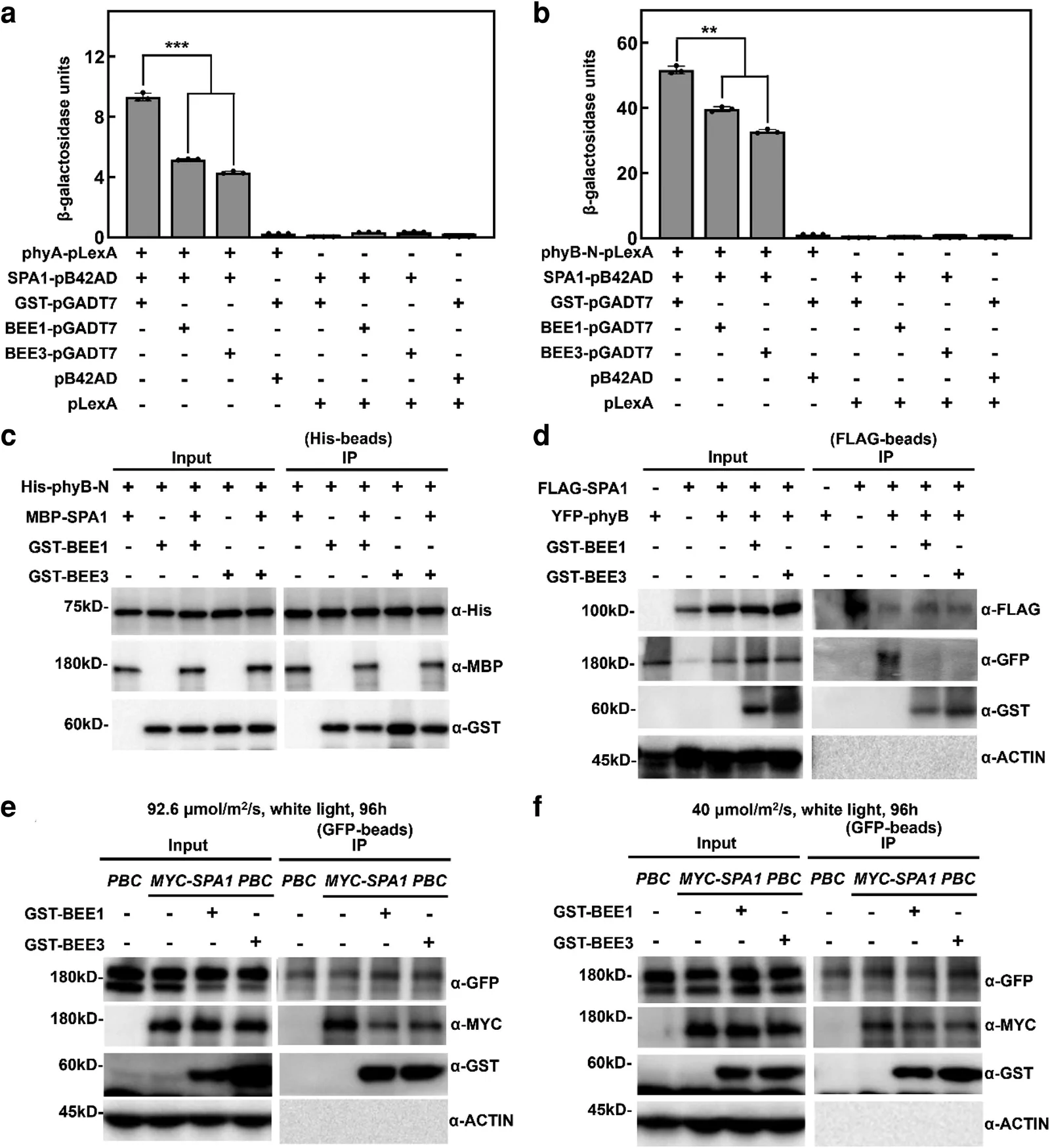
BEE1 and BEE3 repress the interaction between phyA/B and SPA1. (a and b) Yeast three-hybrid assays showing that BEE1 and BEE3 inhibit the interaction of phyA/B with SPA1 in yeast cells. The indicated combinations of phyA, phyB-N, SPA1, BEE1, and BEE3 were expressed in the yeast strain EGY48 (p8oLacZ). The β-galactosidase activities were measures. Error bars represent SD of 3 independent yeast clones. Asterisks represent statistically significant differences (***P < 0.001, *P < 0.05), as determined by one-way ANOVA with Tukey's post hoc analysis. (c) Pull-down assay showing that BEE1 and BEE3 block the interaction between phyB-N and SPA1. The indicated combinations of His-phyB-N, MBP-SPA1, GST-BEE1, and GST-BEE3 were purified in *E. coli*. The precipitated proteins were analyzed by immunoblotting with antibodies against His (CMCTAG), MBP (CMCTAG), and GST (CMCTAG), respectively. (d) Semi-Co-IP assay showing that BEE1 and BEE3 alleviate the interaction between phyB and SPA1. Total proteins were extracted from *N. benthamiana* leaves that transiently expressed 35S:FLAG-SPA1, 35S:YFP-phyB, or a combination of both. These protein extracts were then incubated with or without recombinant GST-BEE1 and GST-BEE3 in the specified binding reactions. The input and precipitated proteins were subjected to immunoblotting with antibodies against FLAG (Sigma-Aldrich), GFP (Abmart), GST (CMCTAG), and ACTIN (Abclonal), respectively. (e and f) Semi-Co-IP assay showing that BEE1 and BEE3 alleviate the interaction between phyB and SPA1. Total proteins were extracted from 4-day old seedlings of *PBC* (*phyB-CFP* Col) and *MYC-SPA1 PBC* homozygous lines grown in the white light of 92.6 μmol/m^2^/s (e) and 40 μmol/m^2^/s (f). The resulting lysates were incubated with recombinant GST-BEE1 or GST-BEE3 in the indicated binding reactions. The input samples and immunoprecipitated proteins were then analyzed by immunoblotting with antibodies against GFP (Abmart), MYC (Sigma-Aldrich), GST (CMCTAG), and ACTIN (Abclonal), respectively.

## Discussion

BEE1, BEE2, and BEE3 belong to the basic helix-loop-helix (bHLH) transcription factors, which were initially discovered as positive regulators of brassinosteroid signaling. Increasing evidence has revealed that BEE1/2/3 also play critical roles in shade avoidance, flowering, and light signaling (Friedrichsen et al. 2002; Yin et al. 2005; Cifuentes-Esquivel et al. 2013; Wang et al. 2019). However, the specific pathways and mechanisms by which BEE1/2/3 mediate these responses remain to be dissected. In the present study, we found that BEE1/2/3 act as photomorphogenic repressors to interact with phyA/B and interfere with the phyA/B–PIF3 association, thereby promoting PIF3 accumulation and its target gene expression. Additional analyses demonstrated that BEE1/2/3 physically interact with SPAs to disrupt the phyA/B–SPAs interaction to further assist to repress plant photomorphogenesis, contributing to avoid excessively activated light signaling and ensure normal photomorphogenic development in plants (Fig. 10).

**Figure 10:**
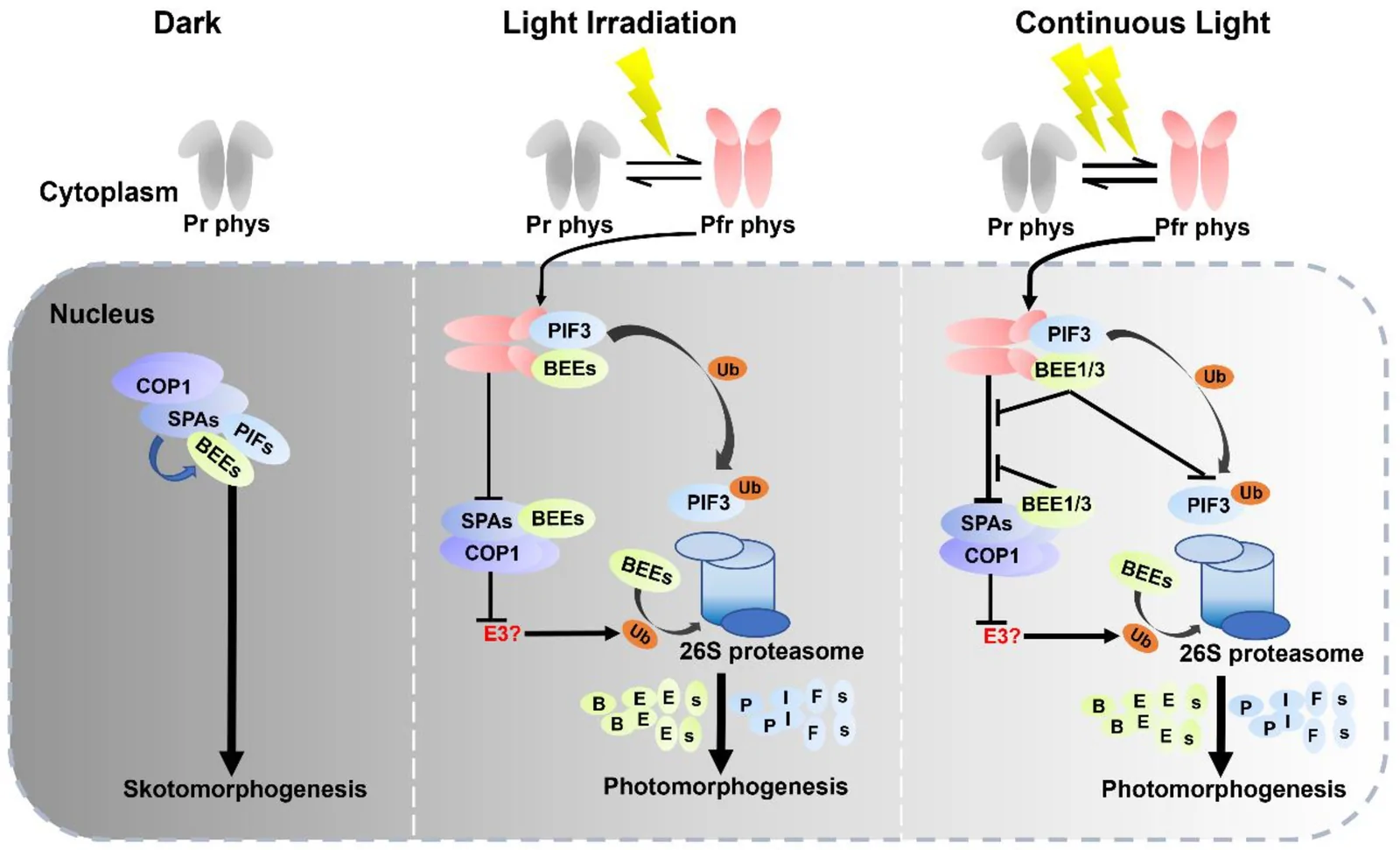
A proposed working model depicting how BEE1/2/3 negatively regulate plant photomorphogenesis. In darkness, BEE1/2/3 transcription factors are stabilized through their interaction with the SPA1-COP1 complex, which protects them from degradation. This allows BEEs to activate gene expression that promotes skotomorphogenesis. Upon light exposure, photoactivated phytochromes (phyA/B) trigger photomorphogenesis via 2 parallel routes: they directly interact with PIF3 and induce its degradation, while concurrently, their interaction with SPAs leads to the disassembly of the repressive COP1-SPA complex, therefore promoting photomorphogenesis. Under continuous high light conditions, however, BEEs play a counterbalancing role by interacting directly with phyA/B. This direct interaction disrupts the formation of the phyA/B-PIF3 complex, which in turn prevents the light-induced degradation of PIF3. The subsequent accumulation of PIF3 suppresses excessive photomorphogenesis. Additionally, the BEE-phyA/B interaction attenuates the phyA/B-SPA1 module that promotes photomorphogenesis, thereby mitigating constitutively active photomorphogenic development. Collectively, these interconnected pathways form a negative feedback loop that fine-tunes and attenuates exaggerated light signaling, ensuring a balanced photomorphogenic response.

Our data indicate that light tightly regulates the transcription and protein stability of BEEs. BEE2 degraded quickly upon light exposure; however, the degradation of BEE1 and BEE3 required continuous light conditions (Fig. 1). Phenotypic analyses showed that *BEE1* and *BEE3* overexpression developed longer hypocotyls, while *BEE2* overexpression did not (Figure S1). These results suggest a functional divergence between BEE1/3 and BEE2. Our screening of BEE1/2/3-interacting factors led to the identification of phyA and phyB as BEE1/2/3-interacting partners. Previous studies have shown that light promotes the phosphorylation and degradation of PIF3, which is the first identified interaction factor of phyB. PIF3 belongs to the bHLH-type transcription factor family and negatively regulates plant photomorphogenesis. Thus, both BEE1/2/3 and PIF3 are bHLH-type transcription factors that interact with phytochromes and negatively regulate plant photomorphogenesis, implying the close similarity between BEE1/2/3 and PIF3. Recent structural studies reported the existence of a 2:1 stoichiometry of phyB-Pfr and PIF (Wang et al. 2024). However, the molar ratio of phyB and BEEs needs further investigation. Importantly, both pull-down and semi-Co-IP assays consistently demonstrate a direct physical interaction between PIF3 and BEE1/3 (Fig. 5a to 5e). Given that both PIF3 and BEE1/3 are bHLH-type transcription factors, their interaction likely facilitates the formation of heterodimers. We propose that these putative BEE-PIF3 heterodimers play a crucial role in repressing photomorphogenesis by modulating the expression of downstream target genes.

Our immunoblot analyses showed that phyA and phyB negatively regulated the protein stability of BEE3. Consistently, BEE1/3 interfere with the interaction between phyA/B and PIF3, which alleviate the degradation of PIF3. Thus, BEE proteins protect PIF3 from degradation to repress photomorphogenesis. However, overexpression of *BEE1* and *BEE3* partially suppressed the long hypocotyl of the *phyA phyB* double mutant under white, red, and far-red light conditions. This observation can potentially be attributed to the involvement of phytohormones, such as brassinosteroids, in this process. Elucidating the role of BEE1/2/3 in the phytohormone action is therefore of great interest.

It is well documented that the COP1–SPAs complex targets photomorphogenesis-promoting factors, such as HY5, LAF1, and BBX21, for ubiquitination and degradation, while stabilizing photomorphogenic repressors like PIF3 (Osterlund et al. 2000; Seo et al. 2003; Xu et al. 2016; Ling et al. 2017; Xu 2020). Whether COP1–SPAs regulate BEE1/2/3 protein abundance remains largely obscure. In this study, our data showed that BEE1/2/3 physically interact with SPA1/4, which in turn promote the stability of BEE1 and BEE3 under both dark and white light conditions. A similar stabilizing role was observed for COP1, which also protects these BEE proteins from degradation across both light regimes. Interestingly, BEE3 was more stable than BEE1 in the *spa1 spa2 spa4* triple mutant, indicating that COP1–SPAs complex may preferentially maintain BEE1 over BEE3. We postulate that these observations may be due to the functional redundancy among SPA proteins and the action of distinct E3 ubiquitin ligases on BEE1 vs BEE3. Consequently, the identity of the specific E3 ubiquitin ligases for BEE1/2/3 has yet to be clarified.

Previous studies have revealed that photoactivated phyA/B and CRY1 translocate into the nucleus and interact with SPAs to accelerate the disassociation of the COP1–SPAs complex, thereby facilitating plant photomorphogenesis (Lian et al. 2011; Liu et al. 2011; Zuo et al. 2011; Lu et al. 2015; Sheerin et al. 2015). Here, we found that BEE1 and BEE3 significantly repress the interaction between phyA/B and SPA1, limiting plant photomorphogenesis under continuous light condition. Thus, this study identifies BEE1 and BEE3 as the first known factors with the novel function of directly impairing the phyA/B-SPA1 interaction. BEE1 has also been proved to interact with CRY1 (Wang et al. 2019). However, it remains to be determined whether BEE1 interferes with the interaction between CYR1 and SPAs. Genetic analysis indicated that the *bee1 bee2 bee3* triple mutant enhanced the constitutively photomorphogenic phenotype of *spa1 spa2 spa4* in the white, blue, and red light. However, the *spa1 spa2 spa4 bee1 bee2 bee3* sextuple mutant was similar to the *spa1 spa2 spa4* triple mutant in the far-red light. These data suggest that SPAs act downstream of BEE1/2/3, consistent with the molecular action by which BEE1/2/3 suppress the interaction between phyA/B and SPAs. In addition, *BEE1* and *BEE3* overexpression partially rescued the short hypocotyl of the *cop1-6* and *spa1 spa2 spa4* mutants, indicating that BEE1 and BEE3 also partially act downstream of COP1 and SPAs, which supports our molecular evidence that COP1-SPAs complexes protect BEE1/3 from degradation. Thus, BEE1/2/3 act upstream of SPAs to inhibit the phyA/B-SPA1 interaction, while also acting downstream of the COP1–SPA complex in a feedback loop that stabilizes BEE1/3 abundance, thereby preventing exaggerated photomorphogenesis under continuous light.

To summarize, our data demonstrate that BEE1/2/3 function as negative regulators of plant photomorphogenesis by working in close concert with phyA/B and SPAs. BEE1/2/3 interact with phyA/B to release PIF3 from phyA/B, contributing to protect PIF3 from degradation and repress photomorphogenesis. Additionally, BEE1/2/3 interact with SPA proteins to interfere with the interaction between phyA/B and SPA1, further indicating that BEE1/2/3 repress plant photomorphogenesis in response to hyperactive light signaling. Thus, BEE1/2/3 are novel photomorphogenic repressors that directly interact with photoreceptors to transduce light signals into the nucleus. This mechanism may serve to fine-tune seedling development via the phyA/B–BEE1/2/3–SPAs module in light signal transduction pathway.

## Materials and methods

### Plant materials and growth conditions

The *Arabidopsis thaliana phyA-211 phyB-9* (Kang et al. 2009), *cop1-6* (McNellis et al. 1994), *spa1 spa2 spa3*, *spa1 spa2 spa4* (Zhu et al. 2008), *YFP-BEE2* Col*, YFP-BEE3* Col (Cao et al. 2022), *phyB-CFP* Col (Chen et al. 2005), *MYC-SPA1* Col (Yang and Wang 2006) are in the Columbia-0 (Col-0) background and were previously described. The *bee1 bee2 bee3* triple mutant in the Col-0 background was kindly provided by Dr. Jaume Martínez García (Friedrichsen et al. 2002; Cifuentes-Esquivel et al. 2013). *Myc-BEE1* transgenic seeds were kindly provided by Dr. Hongtao Liu (Wang et al. 2019). The seeds were surface sterilized with 15% commercial sodium hypochlorite for 5 min, washed 3 times with sterile water, and sown on 1× Murashige and Skoog (MS) medium supplemented with 0.8% agar (Sigma-Aldrich) and 1% sucrose. The seeds were stratified in the dark for 3 days at 4 °C and transferred to white (W) (1.7, 1.93, or 10.35 μmol/m^2^/s), blue (B) (1.06 or 5.21 μmol/m^2^/s), red (R) (15.2, 50.1 or 86.8 μmol/m^2^/s), and far-red (FR) (0.95, 1.68 or 7.98 μmol/m^2^/s) light chambers (Percival) maintained at 22 °C.

### Construction of plasmids

The full-length *BEE1*, *BEE2*, and *BEE3* open reading frame (ORF) were cloned into the *pDONR-221* or *pDONR-223* vector (Invitrogen) and introduced into the plant binary vector *pEarley Gateway 203*, *pEarley Gateway 104, pSPYNE* or *pSPYCE* (Earley et al. 2006) under the 35S promoter using Gateway LR Clonase enzyme mix (Invitrogen). The *pEarley Gateway-MYC-BEE1/2/3*, *pEarley Gateway-YFP-BEE1/2/3, pSPYNE-35S-BEE1*, *pSPYNE-35S-BEE2*, and *pSPYNE-35S-BEE3* constructs were generated. The full-length *phyA*, *phyB*, and *SPA4* ORF were cloned into the *pDONR-223* vector (Invitrogen) and introduced into the plant binary vector *pEarley Gateway 104*, *pEarley Gateway 202*, *pEarley Gateway 203*, *pSPYNE* or *pSPYCE* (Walter et al. 2004; Earley et al. 2006) under the 35S promoter using Gateway LR Clonase enzyme mix (Invitrogen). The *pEarley Gateway-YFP-phyA*, *pEarley Gateway-YFP-phyB*, *pEarley Gateway-YFP-SPA4*, *pEarley Gateway-FLAG-phyB*, *pEarley Gateway-FLAG-SPA1*, *pEarley Gateway-MYC-SPA1*, *pSPYCE-35S-phyA*, *pSPYCE-35S-phyB*, and *pSPYCE-35S-SPA4* constructs were generated. The full-length *phyB* and BEE2 coding sequence (CDS) were cloned into the *pCambia1300-35S-2×FLAG* vector, generating the *pCambia1300-35S-phyB-FLAG* and *pCambia1300-35S-BEE2-FLAG* constructs.

To generate the *pGBKT7-BEE1*, *pGBKT7-BEE1 (1 to 120)*, *pGBKT7-BEE1 (120 to 260)*, *pGBKT7-BEE3*, *pGBKT7-BEE3 (1 to 118)*, and *pGBKT7-BEE3 (118 to 261)* constructs, full-length and truncated *BEE1* and *BEE3* fragments were amplified by PCR with the respective pairs of primers and cloned into the *EcoRI/BamHI* sites of the *pGBKT7* vector (BD Clontech). To generate the *pGBKT7-BEE2*, *pGBKT7-BEE2 (1 to 120)*, *pGBKT7-BEE2 (120 to 304)* constructs, full-length and truncated *BEE2* fragments were amplified by PCR with the respective pairs of primers and cloned into the *EcoRI/SalI* sites of the *pGBKT7* vector (BD Clontech) using a One-step Cloning kit (Vazyme). To generate *pB42AD-BEE1*, *pB42AD-BEE1 (1 to 120)*, and *pB42AD-BEE1 (120 to 260)* constructs, full-length and truncated *BEE1* fragments were amplified by PCR with the respective pairs of primers and cloned into the *EcoRI/XhoI* sites of the *pB42AD* vector (BD Clontech). To generate *pB42AD-BEE2*, *pB42AD-BEE2 (1 to 120)*, *pB42AD-BEE2 (120 to 304)*, *pB42AD-BEE3*, *pB42AD-BEE3 (1 to 118)*, and *pB42AD-BEE3 (118 to 261)* constructs, full-length and truncated *BEE2* and *BEE3* fragments were amplified by PCR with the respective pairs of primers and cloned into the *EcoRI/XhoI* sites of the *pB42AD* vector (BD Clontech) using a One-step Cloning Kit (Vazyme). To generate *pLexA-phyA*, *pLexA-phyA-N*, *pLexA-phyA-C*, *pLexA-phyA-C1*, and *pLexA-phyA-C2* constructs, full-length and truncated *phyA* fragments were amplified by PCR with the respective pairs of primers and cloned into the *EcoRI/XhoI* sites of the *pLexA* vector (BD Clontech) using a One-step Cloning Kit (Vazyme). To generate *pGADT7-phyB*, *pGADT7-phyB-N*, *pGADT7-phyB-C*, *pGADT7-phyB-C1*, and *pGADT7-phyB-C2* constructs, full-length and truncated *phyB* fragments were amplified by PCR with the respective pairs of primers and cloned into the *EcoRI/BamHI* sites of the *pGADT7* vector (BD Clontech) using a One-step Cloning Kit (Vazyme).

To generate *pGEX-4T-1-phyA*, *pMAL-c2x-phyA*, *pET-28a-phyA*, and *pCold TF-phyA* constructs, full-length *phyA* fragments were amplified by PCR with the respective pairs of primers and cloned into the *BamHI/SalI* sites of the *pGEX-4T-1, pMAL-c2x*, *pET-28a*, and *pCold TF* vector. To generate *pGEX-4T-1-SPA1*, *pMAL-c2x-SPA1*, *pET-28a-SPA1*, and *pCold TF-SPA1* constructs, full-length *SPA1* fragments were amplified by PCR with the respective pairs of primers and cloned into the *BamHI/SalI* sites of the *pGEX-4T-1*, *pMAL-c2x*, *pET-28a*, and *pCold TF* vector using a One-step Cloning Kit (Vazyme). To generate *pGEX-4T-1-BEE1, pMAL-c2x-BEE1*, *pET-28a-BEE1*, *pGEX-4T-1-BEE3*, *pMAL-c2x-BEE3*, and *pET-28a-BEE3* constructs, full-length *BEE1* and *BEE3* fragments were amplified by PCR with the respective pairs of primers and cloned into the *EcoRI/SalI* sites of the *pGEX-4T-1, pMAL-c2x,* and *pET-28a* vector. The primers used for plasmid construction are listed in Data Set S1.

### Transgenic plants

To produce the transgenic plants, the *pEarley Gateway-YFP-BEE1, pEarley Gateway-YFP-BEE2*, and *pEarley Gateway-YFP-BEE3* constructs were transformed into *Agrobacterium tumefaciens* GV3101 by the freeze-thaw method and introduced into Col-0 plants via the floral dip method (Clough and Bent 1998). Transgenic plants were selected on MS medium containing 20 mg/L Basta.

### Measurement of hypocotyl length

To measure the hypocotyl length of seedlings, seeds were sown on plates and stratified at 4 °C in the dark for 3 days and kept in continuous white light for 8 hours in order to induce uniform germination. The seeds were transferred to dark, white, blue, red, and far-red light conditions and incubated at 22 °C for 4 days (Heng et al. 2019). The hypocotyls length of the seedlings were measured using ImageJ software.

### Co-immunoprecipitation assays and immunoblot analysis

For co-immunoprecipitation (Co-IP) assays using *Nicotiana benthamiana* leaves, *Agrobacterium* strain GV3101 carrying the *35S:YFP-phyA*/*SPA4* alone or together with *35S:MYC-BEE1/2/3* constructs were transiently infiltrated into *N. benthamiana* leaves. *Agrobacterium* strain GV3101 carrying the *35S:phyB-FLAG* alone or together with *35S:YFP-BEE1/2/3* constructs were transiently infiltrated into *N. benthamiana* leaves. The plants were grown under long-day conditions for 2 d. Leaves were then harvested and lysed in Co-IP buffer containing 50 mM Tris-HCl (pH 7.5), 150 mM NaCl, 1 mM EDTA, 10% glycerol, 0.05 to 0.1% Tween20, 1 tablet/50 mL Roche protease inhibitor cocktail, and 1 mM phenylmethylsulfonyl fluoride (PMSF). Note: Protease inhibitors (PMSF and cocktail) were added to the buffer immediately before use. The extracts were incubated with 20 μL of anti-GFP beads (Alpalife), anti-MYC beads (Alpalife), or anti-FLAG beads (Alpalife) for 4 h at 4 °C. The GFP/MYC/FLAG-beads were washed 3 times with protein extraction buffer. The precipitates were eluted by adding 20 μL 2× sodium dodecyl sulfate (SDS) loading buffer, boiled at 100 °C for 10 min, and analyzed by immunoblot analysis. MYC-BEE1, MYC-BEE2, and MYC-BEE3 proteins were detected with an α-c-MYC antibody (Sigma-Aldrich). BEE2-FLAG and phyB-FLAG were detected with an α-FLAG antibody (Sigma-Aldrich). YFP-phyA, YFP-SPA4, and YFP-BEE1/2/3 were detected with an α-GFP antibody (Abmart).

For immunoblot analysis, wild-type, mutant, and transgenic Arabidopsis seedlings were homogenized in protein extraction buffer containing 100 mM NaH_2_PO_4_, 10 mM Tris·HCl, 200 mM NaCl, 8 M Urea, pH 8.0, 1 mM PMSF and 1× complete protease inhibitor cocktail (Roche). Primary antibodies used in this study were anti-c-MYC (Sigma-Aldrich), anti-FLAG (Sigma-Aldrich), anti-GFP (Abmart), anti-PIF3 (a gift from Dr. Jian Li, 1:1,000 dilution), anti-ACTIN (ABclonal), and anti-HSP82 (Beijing Protein Innovation).

### Yeast two-hybrid and modified yeast three-hybrid assays

The yeast two-hybrid assays with the Gal4 system were performed using the Matchmaker GAL4 Two-Hybrid System (BD Clontech). The respective combinations of pGADT7 and pGBKT7 fusion plasmids were cotransformed into yeast strain AH109. The empty pGADT7 and pGBKT7 vectors were cotransformed in parallel as negative controls. To assess protein interactions, the transformed yeast cells were grown on SD/-3 (-His/-Leu/-Trp) and SD/-4 (-Ade-His/-Leu/-Trp) dropout media (Coolaber). The interactions were observed after 3 d of incubation at 30 °C. Yeast transformation was performed as described in the Yeast Protocols Handbook (BD Clontech and Coolaber).

The yeast two-hybrid assay with the pLexA system were described previously and performed using the Matchmaker LexA Two-Hybrid System (BD Clontech) (Li et al. 2023). The respective combinations of pB42AD and plexA fusion plasmids were cotransformed into yeast strain EGY48 (p8oLacZ). The empty pB42AD and pLexA vectors were cotransformed in parallel as negative controls. To assess protein interactions, the transformed yeast cells were grown on SD/-3 (-Trp/-His/-Ura/) dropout medium. The interactions were observed after 3 d of incubation at 30 °C on SD/-3 (-Trp/-His/-Ura/)+X-Gal (Coolaber). Yeast transformation was performed as described in the Yeast Protocols Handbook (BD Clontech and Coolaber). The β-galactosidase activity was measured using a Yeast β-Galactosidase Assay Kit (Thermo scientific 75,768).

For the modified yeast three-hybrid assay, the respective combinations of AD and BD fusion proteins were cotransformed into yeast strain EGY48 (p8oLacZ), and transformants were selected and grown on SD/-Trp-His-Ura-Leu dropout medium (Coolaber). Yeast transformation and liquid assay were conducted as described in the Yeast Protocols Handbook (BD Clontech). The β-galactosidase activity was measured using a Yeast β-Galactosidase Assay Kit (Thermo scientific 75,768) (Li et al. 2023).

### Bimolecular fluorescence complementation assays

For the bimolecular fluorescence complementation (BiFC) assays, the YFP^N^– and YFP^C^– fused plasmids were transformed into *Agrobacterium* strain GV3101, and the indicated transformant pairs were infiltrated into *N. benthamiana* leaves (Walter et al. 2004). A Carl Zeiss confocal laser scanning microscope (LSM880) was used to detect the YFP fluorescence signals. YFP fluorescence was excited by a 514 nm laser and detected between 517 and 589 nm.

### Preparation of recombinant proteins

The recombinant proteins were prepared as described previously (Song et al. 2024a). All constructs were transformed into *Escherichia coli* BL21 (DE3), and then the cells were treated with 0.3 mmol/L isopropyl-β-D-thiogalactoside and incubated overnight at 4 °C or 16 °C to induce the expression of the fusion proteins. The cells were collected by centrifugation at 4,000 rpm for 10 min at 4 °C. The MBP fusion proteins were purified with MBPSep Dextrin Agarose Resin 6FF (Yeasen Biotechnology), the GST fusion proteins were purified with GSTSep Glutathione Agarose Resin (Yeasen Biotechnology), and the His fusion proteins were purified with HisSep Ni-NTA Agarose Resin (Yeasen Biotechnology).

### In vitro pull-down assays

In vitro pull-down assays were described previously with minor modifications (Lin et al. 2017), 1 μg of purified recombinant bait proteins (MBP-PIF3), 1 μg of purified recombinant prey proteins (His-TF-phyA and His-phyB-N), and 1 μg of purified recombinant GST-BEE1 and GST-BEE3 proteins were added to 1 mL of binding buffer (20 mM Tris-HCl pH7.5, 200 mM NaCl, 10% glycerol, 0.1% Triton X-100, and 1 mM PMSF). After incubation for 2 h at 4 °C, amylose resins (Yeasen Biotechnology) were added and incubated for a further 2 h, and then washed 3 times with the binding buffer. The pulled-down proteins were eluted with 5× SDS loading buffer at 95 °C for 10 min. The protein samples were subjected to 10% SDS-PAGE. Input and pull-down prey proteins were detected by immunoblotting using anti-MBP (CMCTAG), anti-GST (CMCTAG), and anti-His (CMCTAG) antibodies.

### Semi-Co-IP assays

For semi-Co-IP assays using *Nicotiana benthamiana* leaves, *Agrobacterium* strain GV3101 carrying the *35S:FLAG-SPA1*, *35S:YFP-phyB*, or their combination constructs were transiently infiltrated into *N. benthamiana* leaves. The plants were grown under long-day conditions for 3 d and the leaves lysed with Co-IP buffer. The extracts were incubated with 20 μL of anti-FLAG beads (Alpalife) with or without purified GST-BEE1/3 for 4 h at 4 °C. The FLAG-beads were washed 3 times with protein extraction buffer. The precipitates were eluted by adding 20 μL 2× SDS loading buffer, boiled at 100 °C for 10 min, and analyzed by immunoblot analysis. FLAG-SPA1 proteins were detected with an α-FLAG antibody (Sigma-Aldrich). YFP-phyB were detected with an α-GFP antibody (Abmart). GST-BEE1 and GST-BEE3 were detected with an α-GST antibody (CMCTAG).

Seedlings of *PBC* (*phyB-CFP* Col) and *MYC-SPA1 PBC*, grown under white light for 4 days, were harvested and lysed with Co-IP buffer. The resulting protein extracts were incubated with 20 μL of anti-GFP beads (Alpalife) for 4 h at 4 °C, in the presence or absence of recombinant GST-BEE1/3. The GFP beads were washed 3 times with protein extraction buffer. The precipitates were eluted with 20 μL of 2× SDS loading buffer by boiling at 100 °C for 10 min. The input and immunoprecipitated samples were subjected to immunoblotting using antibodies against GFP (Abmart), MYC (Sigma-Aldrich), GST (CMCTAG), and ACTIN (Abclonal), respectively.

### Cell-free protein degradation assays

Cell-free protein degradation assays were conducted as described previously (Osterlund et al. 2000; Song et al. 2023; Fang et al. 2024). Col, *bee1 bee2 bee3*, *cop1-6*, and *spa1 spa2 spa4* seedlings were grown in the dark or white light for 4 days. Total proteins were extracted at 4 °C for 30 min by degradation buffer (25 mM Tris-HCl pH7.5, 10 mM NaCl, 10 mM MgCl_2_, 4 mM PMSF, 5 mM DTT, and 10 mM ATP). Protein concentration was detected by Bio-Rad protein assay. About 100 μg plant extracts and 200 ng of recombinant MBP-PIF3, GST-BEE1, and GST-BEE3 proteins were incubated at 22 °C in the dark or white light for the indicated times. Then, immunoblot analysis was performed to detect the protein amounts using anti-MBP (CMCTAG), anti-GST (CMCTAG), anti-ACTIN (Abclonal), and anti-HSP82 (Beijing Protein Innovation) antibodies.

### RNA extraction and RT-qPCR

Total RNA was extracted from 4-d-old Arabidopsis seedlings grown under white light using an RNeasy plant mini kit (Omega). cDNAs were synthesized from 2 μg of total RNA using the Hifair II 1st Strand cDNA synthesis Super Mix (Yeasen Biotechnology) according to the manufacturer's instructions. The cDNAs were subjected to RT-qPCR using the Step One Plus Real-time PCR Detection System (Applied Biosystems) and Hieff qPCR SYBR Green Master Mix (Yeasen Biotechnology). PCR was performed in triplicate for each sample, and the expression levels were normalized to that of the *PP2A* or *ACTIN2* gene (Song et al. 2024b). The primers used in this study are listed in Data Set S1.

### RNA sequencing

Total RNA was extracted from the 4-day-old Col, *bee1 bee2 bee3*, *spa1 spa2 spa4*, and *phyA phyB* seedlings grown in constant red light (120.1 μmol/m^2^/s) conditions. mRNA sequencing libraries were constructed, and sequencing was performed using the T7 sequencer from BGI (Beijing Genomics Institute, China). Three independent biological replicates were performed in which the wild-type, *bee1 bee2 bee3*, *spa1 spa2 spa4*, and *phyA phyB* were sampled independently. RNA-seq data analysis was performed following the previously described method (Sun et al. 2020). Briefly, adapter sequences and low-quality reads were removed using fastp (Chen et al. 2018) with the option-length_required 70. The resulting clean reads were mapped to the *Arabidopsis* reference genome (TAIR10, https://www.arabidopsis.org) using STAR (Dobin et al. 2013) with the parameter--outFilterMultimapNmax 10. Read counts for genes were obtained using featureCounts (Liao et al. 2014) with the -p option to count fragments instead of reads. The count data were then subjected to differential expression analysis using DESeq2 (Love et al. 2014), with significance thresholds set at log_2_fold change>1 and *P* < 0.05. Venn diagrams were generated with the VennDiagram package (Chen and Boutros 2011), heatmaps with pheatmap (Kolde 2019), and scatter plots with ggplot2 (Wickham 2016). GO enrichment analysis was performed using clusterProfiler (Wu et al. 2021) with the org.At.tair.db annotation database, and the resulting dot plots were produced with ggplot2.

### Statistical analysis

For immunoblot quantification, the relative band intensities were measured with ImageJ from 3 independent experiments. Differences between 2 groups were analyzed using a two-sided Student's *t* test, whereas one-way ANOVA followed by Tukey's post hoc test was employed for multiple comparisons. Data for quantitative analyses are presented as mean ± SD. Statistical analyses were performed using GraphPad Prism software (version 8.0) and Microsoft Excel.

## Accession numbers

Sequence data from this article can be found in the Arabidopsis TAIR database (www.arabidopsis.org) or the GenBank/EMBL libraries under the following accession numbers: BEE1 (AT1G18400), BEE2 (AT4G36540), BEE3 (AT1G73830), phyA (AT1G09570), phyB (AT2G18790), PIF3 (AT1G09530), COP1 (AT2G32950), SPA1 (AT2G46340), SPA2 (AT4G11110), SPA3 (AT3G15354), SPA4 (AT1G53090). RNA-seq data were submitted to the National Center for Biotechnology Information Sequence Read Archive (https://submit.ncbi.nlm.nih.gov/subs/sra/) under accession number PRJNA1502772.

## Supplementary Material

koag284_Supplementary_Data

## Data Availability

All data generated and analyzed in this study are available within the article and its Supplementary Information files. The data are available from the corresponding author upon reasonable request.
