# Supplementary material for: BR-ENHANCED EXPRESSION 1/2/3 fine-tune plant photomorphogenesis by regulating the phyA/B-SPA1 cascade
Source: Plant Cell. 2026 Sep 21;38(10):koag284. doi: 10.1093/plcell/koag284 (PMC13674110; doi:10.1093/plcell/koag284)
Supplement: Supplementary file 1 [file koag284_supplementary_data.docx]

**Supplementary Figures**

**Supplementary Figure S1. BEE1, BEE2, and BEE3 are photomorphogenic repressors.** **(Supports Figure 1)**

**(A-H)** Hypocotyl lengths and phenotypes of Col, *bee1 bee2 bee3, MYC-BEE1* Col*,* *YFP-BEE2* Col*,* and *YFP-BEE3* seedlings grown in W (10.35 μmol/m^2^/s) **(A and B)**, B (5.21 μmol/m^2^/s) **(C and D)**, R (86.8 μmol/m^2^/s) **(E and F)**, and FR (7.98 μmol/m^2^/s) **(G and H)** light conditions. Hypocotyl length is expressed in mm. Data are means ± SE; n≥20. Different letters above the bars indicate significant differences (*P* < 0.05), as determined by one-way ANOVA with Tukey’s post-hoc analysis. The experiments were performed three times with similar results. The graphs depict one of three experiments.

**Supplementary Figure S2. Light regulates *BEE1/2/3* gene expression. (Supports Figure 1)**

**(A-C)** *BEE1, BEE2* and *BEE3* transcript levels in 4-d-old Col grown in the dark and transferred to white light (92.65 μmol/m^2^/s) for the indicated time (****P* < 0.001). Error bars represent SD of three technical replicates.

**Supplementary Figure S3. BEE1/2/3 interact with phyB. (Supports Figure 2)**

Yeast two-hybrid assay of interactions between the indicated phyB and BEE1/2/3 in the Gal4 system. A series of truncation fragments of phyB were fused to GAL4 DAD in pGADT7. The N-terminus and C-terminus of BEE1/2/3 were fused to GAL4 DBD in pGBKT7. AH109 yeast strains containing the indicated vectors were grown on SD/-Leu-Trp-Ade-His for 3 days.

**Supplementary****Figure S4. *BEE1/3* act partially downstream of *PHYA/B*.** **(Supports Figure 4)**

**(A-H)** Hypocotyl lengths and phenotypes of 4-d-old Col, *phyA phyB*, *MYC-BEE1 phyA phyB, YFP-BEE3 phyA phyB, MYC-BEE1* Col*,* and *YFP-BEE3* Col seedlings grown in W (10.35 μmol/m^2^/s) **(A and B)**, B (5.21 μmol/m^2^/s) **(C and D)**, R (86.8 μmol/m^2^/s) **(E and F)**, and FR (7.98 μmol/m^2^/s) **(G and H)** light conditions. Hypocotyl length is expressed in mm. Data are means ± SE; n≥20. Different letters above the bars indicate significant differences (*P* < 0.05), as determined by one-way ANOVA with Tukey’s post-hoc analysis. The experiments were performed three times with similar results. The graphs depict one of three experiments. (****P* < 0.001, **P* < 0.05), as determined by one-way ANOVA with Tukey’s post-hoc analysis.

**Supplementary Figure S5. phyA and phyB promote the protein degradation of BEE3. (Supports Figure 4)**

Immunoblot assay showing YFP-BEE3 protein levels in 4-d-old Col, *YFP-BEE3* Col*,* and *YFP-BEE3 phyA phyB* grown in white (92.65 μmol/m^2^/s) and red light (109.9 μmol/m^2^/s). YFP-BEE3 proteins were detected with α-GFP antibody (Abmart). ACTIN served as a loading control.

**Supplementary Figure S6. BEE1/2/3 do not regulate the gene expression of *PIF1/3/4/5*. (Supports Figure 5)**

**(A-D)** *PIF1*, *PIF3*, *PIF4*, and *PIF5* transcript levels in 4-d-old Col, *bee1 bee2 bee3,* and *pifq* seedlings grown in the dark and red light (109.9 μmol/m^2^/s). Error bars represent SD of three technical replicates.

**Supplementary Figure S7. *PIFs* are epistatic to *BEE1/2/3* in the dark. (Supports Figure 5)**

**(A-H)** Hypocotyl lengths and phenotypes of Col, *bee1 bee2 bee3, pifq,* and *bee1 bee2 bee3 pifq* seedlings grown in the dark **(A and B),** W (10.35 μmol/m^2^/s) **(C and D)**, B (5.21 μmol/m^2^/s) **(E and F)**, R (86.8 μmol/m^2^/s) **(G and H)**, and FR (7.98 μmol/m^2^/s) **(I and J)** light conditions. Hypocotyl length is expressed in mm. Data are means ± SE; n≥20. Different letters above the bars indicate significant differences (*P* < 0.05), as determined by one-way ANOVA with Tukey’s post-hoc analysis. The experiments were performed three times with similar results. The graphs depict one of three experiments.

**Supplementary Figure S8.** **SPA1 interacts with BEE1/2/3. (Supports Figure 6)**

**(A)** Diagram of truncated SPA1/ and SPA4. **(B)** Yeast two-hybrid assay of interactions between the indicated BEE1/2/3 and SPA1/4 in the pLexA system. A series of truncation fragments of SPA1 and SPA4 were introduced into pLexA. The N-terminus and C-terminus of BEE1/2/3 were introduced into pB42AD. EGY48 (p8oLacZ) yeast strains containing the indicated vectors were grown on either SD/-Trp-His-Ura or SD/Raf/Gal-Trp-His-Ura with X-Gal. **(C)** BiFC assay detecting the interaction between BEE1/2/3 and SPA1. Full-length BEE1/2/3 and SPA4 were fused to the split N- or C-terminal (YFP^N^ or YFP^C^) fragments of YFP. Unfused YFP N-terminal (YFP^N^) or YFP C-terminal (YFP^C^) fragments and AGO6-YFP^C^ were used as negative controls. Merge, merged images of YFP channel and bright field. Bar = 50 μm. **(D-F)** Co-IP analysis showing that FLAG-SPA1 interacts with YFP-BEE1/2/3. Total proteins were extracted from *N. benthamiane* leaves transiently expressing 35S:FLAG-SPA1 alone or together with 35S:YFP-BEE1/2/3. YFP-BEE1 (**D**), YFP-BEE2 (**E**), and YFP-BEE3 (**F**) proteins were immunoprecipitated with α-GFP beads (Alpalife), and the co-immunoprecipitated FLAG-SPA1 were detected with an α-FLAG antibody (Sigma-Aldrich).

**Supplementary Figure S9. Mutations in *BEE1/2/3* enhance the constitutively photomorphogenic phenotype of *spa1 spa2 spa4*. (Supports Figure 7)**

**(A-H)** Hypocotyl lengths and phenotypes of 4-d-old Col, *bee1 bee2 bee3*, *spa1 spa2 spa4, bee1 bee2 bee3 spa1 spa2 spa4, spa1 spa2 spa3*, and *bee1 bee2 bee3 spa1 spa2 spa3* seedlings grown under far-red light at three low intensity: 0.52 μmol/m^2^/s **(A, B)**, 0.317 μmol/m^2^/s **(C, D)**, 0.138 μmol/m^2^/s) **(E, F)**. Hypocotyl length is expressed in mm. Data are means ± SE; n≥20. Different letters above the bars indicate significant differences (*P* < 0.05), as determined by one-way ANOVA with Tukey’s post-hoc analysis. The experiments were performed three times with similar results. The graphs depict one of three experiments.

**Supplementary Figure S10.** **Overexpression of *BEE1* and *BEE3* partially rescue the hypocotyl length of *spa1 spa2 spa4* in the light.** **(Supports Figure 7 and Figure 8)**

**(A-F)** Hypocotyl lengths and phenotypes of 4-d-old Col, *spa1 spa2 spa4*, *MYC-BEE1 spa1 spa2 spa4, YFP-BEE3 spa1 spa2 spa4, MYC-BEE1* Col*,* and *YFP-BEE3* Col seedlings grown in B (1.06 μmol/m^2^/s) **(A and B)**, R (50.1 μmol/m^2^/s) **(C and D)**, and FR (1.68 μmol/m^2^/s) **(E and F)** light conditions. Hypocotyl length is expressed in mm. Data are means ± SE; n≥20. Different letters above the bars indicate significant differences (*P* < 0.05), as determined by one-way ANOVA with Tukey’s post-hoc analysis. The experiments were performed three times with similar results. The graphs depict one of three experiments.

**Supplementary Figure S****11. Overexpressing *BEE1* and *BEE3* partially rescue the hypocotyl length of *cop1-6* in the light.** **(Supports Figure 7 and Figure 8)**

**(A-F)** Hypocotyl lengths and phenotypes of 4-d-old Col, *cop1-6*, *MYC-BEE1 cop1-6, YFP-BEE3 cop1-6, MYC-BEE1* Col*, YFP-BEE3* Col seedlings grown in B (1.06 μmol/m^2^/s) **(A and B)**, R (15.2 μmol/m^2^/s) **(C and D)**, and FR (1.68 μmol/m^2^/s) **(E and F)** light conditions. Hypocotyl length is expressed in mm. Data are means ± SE; n≥20. Different letters above the bars indicate significant differences (*P* < 0.05), as determined by one-way ANOVA with Tukey’s post-hoc analysis. The experiments were performed three times with similar results. The graphs depict one of three experiments.

**Supplementary Figure S12. BEE1/2/3, phyA/B, and SPA1/2/4 regulate hypocotyl elongation-related genes. (Supports Figure 7 and Figure 8)**

**(A)** Venn diagram shows the overlaps between BEE1/2/3-, phyA/B-, and SPA1/2/4-regulated genes. **(B)** Venn diagram shows the genes repressed by phyA/B but activated by BEE1/2/3 and SPA1/2/4. **(C)** Venn diagram shows the genes activated by phyA/B but repressed by BEE1/2/3 and SPA1/2/4. **(D)** Heatmap analysis of BEE1/2/3-, phyA/B-, and SPA1/2/4-regulated genes. The bar represents the row z-score. **(E)** Correlation by scatterplot of log2 fold change values between BEE1/2/3- and phyA/B-regulated genes. **(F)** Correlation by scatterplot of log2 fold change values between BEE1/2/3- and SPA1/2/4-regulated genes. **(G-I)** GO functional categorization of BEE1/2/3 **(G)-**, phyA/B **(H)-** and SPA1/2/4 **(I)-**regulated genes. Rich ratio indicates the ratio of the number of candidate genes to the total number of genes assigned to this GO term.

**Supplementary Figure S13. COP1-SPAs complex promotes the protein accumulation of BEE1 and BEE3. (Supports Figure 8)**

**(A and B)** Degradation of recombinant GST-BEE1 and GST-BEE3 in the cell-free system. Protein extracts were prepared from 4-d-old Col and *spa1 spa2 spa4* seedling in the white light and then incubated with GST-BEE1 **(A**) and GST-BEE3 **(B)** over the indicated time course. HSP82 was used as a nondegraded loading control. **(C and D)** Degradation of recombinant GST-BEE1 and GST-BEE3 in the cell-free system. Protein extracts were prepared from 4-d-old Col and *cop1-6* seedling in the white light and then incubated with GST-BEE1 **(C)** and GST-BEE3 **(D)** over the indicated time course. ACTIN was used as a nondegraded loading control.

**Supplementary Data Set 1. List of primers used in this study.**

| **Primer name** | **Primer sequence (5’→3’)** | |
| --- | --- | --- |
| **Construct** | **Underscored nucleotides indicate restriction sites for cloning** | **Construct name** |
| BEE1-attB1 | GGGGACAAGTTTGTACAAAAAAGCAGGCT**GG**ATGGCAAATTTCGAGAATCTTTCT | pDonR223-BEE1 |
| BEE1-attB2 no stop | GGGGACCACTTTGTACAAGAAAGCTGGGT**G**AAGGGACCATGTTGATAAATGGAA |  |
| BEE2-attB1 | GGGGACAAGTTTGTACAAAAAAGCAGGCT**GG**ATGGACTTGTCTGTACTTGATAGGC | pDonR223-BEE2 |
| BEE2-attB2 no stop | GGGGACCACTTTGTACAAGAAAGCTGGGT**G**CTTGAGGCTGAAGAAATTGGAAAC |  |
| BEE3-attB1 | GGGGACAAGTTTGTACAAAAAAGCAGGCT**GG**ATGGCGAATCTCTCTTCTGATT | pDonR223-BEE3 |
| BEE3-attB2 no stop | GGGGACCACTTTGTACAAGAAAGCTGGGT**G**AAGGGTCCACGATGATGAATG |  |
| PHYA-N-attB1 | GGGGACAAGTTTGTACAAAAAAGCAGGCT**CC**ATGTCAGGCTCTAGGCCGA | pDonR223-phyA |
| PHYA-C/C2-attB2-3369bp | GGGGACCACTTTGTACAAGAAAGCTGGGT**C**CTTGTTTGCTGCAGCGAG |  |
| PHYB-N-attB1 | GGGGACAAGTTTGTACAAAAAAGCAGGCT**CC**ATGGTTTCCGGAGTCGG | pDonR223-phyB |
| PHYB-C/C2-attB2-3519bp | GGGGACCACTTTGTACAAGAAAGCTGGGT**C**ATATGGCATCATCAGCATCAT |  |
| SPA4-pDonr223-F | GGGGACAAGTTTGTACAAAAAAGCAGGCT**CT**ATGAAGGGTTCTTCAGAATCTAGTTC | pDonR223-SPA4 |
| SPA4-pDonr223-R-2385bp | GGGGACCACTTTGTACAAGAAAGCTGGGT**C**TACCATCTCCAAAATCTTGATATTGC |  |
| pB42AD-BEE1-EcoRI-F | GATTATGCCTCTCCCGAATTCATGGCAAATTTCGAGAATCTTTCT | pB42AD-BEE1,  pB42AD-BEE1  (1-120),  pB42AD-BEE1  (120-260) |
| pB42AD-BEE1-120aa-XhoI-R-360bp-stop | CCGCTCGAGTCAACCTGTTTCAGTGGCAATAACATT |  |
| pB42AD-BEE1-120aa-EcoRI-F-358bp | CCGGAATTCATGGGTTCTTTGAGAAGAGGTAAGAGG |  |
| pB42AD-BEE1-XhoI-R-783bp | CCGCTCGAGTCAAAGGGACCATGTTGATAAATG |  |
| pB42AD-BEE2-EcoRI-F | GCCTCTCCCGAATTCATGGACTTGTCTGTACTTGATAGGC | pB42AD-BEE2,  pB42AD-BEE2  (1-120),  pB42AD-BEE2  (120-304) |
| pB42AD-BEE2-120aa-XhoI-R-pB42AD-360bp-stop | CCAAAGCTTCTCGAGTTACCCTTTCATGCTTGGCTCT |  |
| pB42AD-BEE2-120aa-EcoRI-F-340bp | GCCTCTCCCGAATTCATGACAGAGCCAAGCATGAAAGG |  |
| pB42AD-BEE2-XhoI-R-915bp | AGAAGTCCAAAGCTTCTCGAGTTACTTGAGGCTGAAGAAATTGGAAAC |  |
| pB42AD-BEE3-EcoR I-F | GATTATGCCTCTCCCGAATTCATGGCGAATCTCTCTTCTGATT | pB42AD-BEE3,  pB42AD-BEE3  (1-118),  pB42AD-BEE1  (118-261) |
| pB42AD-BEE3-118aa-XhoI-R-354bp-stop | CCAAAGCTTCTCGAGTCATCCATTTATGGAAACTTGAGCA |  |
| pB42AD-BEE3-118aa-EcoRI-F-331bp | GCCTCTCCCGAATTCATGTCTGCTCAAGTTTCCATAAATGG |  |
| pB42AD-BEE3-XhoI-R | AGAAGTCCAAAGCTTCTCGAGTCAAAGGGTCCACGATGATG |  |
| pLexA-BEE1-EcoRI-F | AACGGCGACTGGCTGGAATTCATGGCAAATTTCGAGAATCTTTCT | pLexA-BEE1 |
| pLexA-BEE1-XhoI-R | TTGGCTGCAGGTCGACTCGAGTCAAAGGGACCATGTTGATAAATG |  |
| pLexA-BEE2-EcoRI-F | AACGGCGACTGGCTGGAATTCATGGACTTGTCTGTACTTGATAGGC | pLexA-BEE2 |
| pLexA-BEE2-XhoI-R | TTGGCTGCAGGTCGACTCGAGTTACTTGAGGCTGAAGAAATTGGAAAC |  |
| pLexA-BEE3-EcoRI-F | AACGGCGACTGGCTGGAATTCATGGCGAATCTCTCTTCTGATT | pLexA-BEE3 |
| pLexA-BEE3-XhoI-R | TTGGCTGCAGGTCGACTCGAGTCAAAGGGTCCACGATGATG |  |
| pGAD/GBK-BEE1-EcoRI-F | CCGGAATTCATGGCAAATTTCGAGAATCTTTC | pGBKT7-BEE1,  pGBKT7-BEE1  (1-120),  pGBKT7-BEE1  (120-260) |
| pGAD/GBK-BEE1-120aa-BamHI-R | CGCGGATCCACCTGTTTCAGTGGCAATAACATT |  |
| pGAD/GBK-BEE1-120aa-EcoRI-F | CCGGAATTCATGAATGTTATTGCCACTGAAACAGGT |  |
| pGAD/GBK-BEE1-BamHI-R | CGCGGATCCAAGGGACCATGTTGATAAATGGA |  |
| pGBKT7-BEE2-EcoRI-F | ATGGAGGCCGAATTCATGGACTTGTCTGTACTTGATAGGC | pGBKT7-BEE2,  pGBKT7-BEE2  (1-120),  pGBKT7-BEE2  (120-304) |
| pGBK-BEE2-120aa-SalI-R | CCGCTGCAGGTCGACCCCTTTCATGCTTGGCTCTGT |  |
| pGBKT7-BEE2 120-304aa-EcoRI-F | ATGGAGGCCGAATTCATGACAGAGCCAAGCATGAAAGGG |  |
| pGBKT7-BEE2-SalI-R | CCGCTGCAGGTCGACTTACTTGAGGCTGAAGAAATTGGAAAC |  |
| pGAD/GBK-BEE3-EcoRI- | CCGGAATTCATGGCGAATCTCTCTTCTGATTTT | pGBKT7-BEE3,  pGBKT7-BEE3  (1-120),  pGBKT7-BEE3  (120-261) |
| pGAD/GBK-BEE3-118aa-BamHI-R | CGCGGATCCTCCATTTATGGAAACTTGAGCAGA |  |
| pGAD/GBK-BEE3-118aa-EcoRI-F | CCGGAATTCATGTCTGCTCAAGTTTCCATAAATGGA |  |
| pGAD/GBK-BEE3-BamHI-R | CGCGGATCCAAGGGTCCACGATGATGAATG |  |
| pLexA-PHYA-N-EcoRI-F | GACTGGCTGGAATTCATGTCAGGCTCTAGGCCGA | pLexA-phyA,  pLexA-phyA-N,  pLexA-phyA-C,  pLexA-phyA-C1,  pLexA-phyA-C2 |
| pLexA-PHYA-N-XhoI-R-1851bp | GCAGGTCGACTCGAGAGCTTCTAGTTCTTGTATACCATCAATT |  |
| pLexA-PHYA-C/C1-EcoRI-F-1687bp | GACTGGCTGGAATTCATGACAAGGAGTTTACCTTGGAAGGACTAT |  |
| pLexA-PHYA-C/C2-XhoI-R-3369bp | GCAGGTCGACTCGAGCTTGTTTGCTGCAGCGAGTT |  |
| pLexA-PHYA-C1-XhoI-R-2553bp | GCAGGTCGACTCGAGACACTCCACATACTTGCCACCTC |  |
| pLexA-PHYA-C2-EcoR I-F-2551bp | GACTGGCTGGAATTCATGTGTCTGTTGTGTGTGAGTAAGAAACT |  |
| pGADT7-PHYB-N-EcoRI-F | GAGGCCAGTGAATTCATGGTTTCCGGAGTCGG | pGADT7-phyB,  pGADT7-phyB-N,  pGADT7-phyB-C,  pGADT7-phyB-C1,  pGADT7-phyB-C2 |
| pGADT7-PHYB-N-BamHI-R-1959bp | GAGCTCGATGGATCCTGCAACTGCACCTAACTCATCA |  |
| pGADT7-PHYB-C/C1-EcoRI-F-1798bp | GAGGCCAGTGAATTCATGGAAACTGCGGAAATGGATG |  |
| pGADT7-PHYB-C/C2-BamHI-R-3519bp | GAGCTCGATGGATCCATATGGCATCATCAGCATCATG |  |
| pGADT7-PHYB-C1- BamHI-R-2730bp | GAGCTCGATGGATCCCTCAGGGCTCGGGATTTG |  |
| pGADT7-PHYB-C2-EcoR I -F-2731b | GAGGCCAGTGAATTCATGCTGCAGCAAGCTTTAGCAGTC |  |
| pGEX/pMAL-c2x/ pET-28a/pCold TF-PHYA-BamHI-F | CGGGATCCATGGAGAAAAAAATGTCAGGCT | pGEX-4T-1-phyA,  pMAL-c2x-phyA,  pET-28a-phyA,  pCold TF-phyA |
| pGEX/pMAL-c2x/ pET-28a/pCold TF-PHYA-SalI-R-3366bp | GCGTCGACCTTGTTTGCTGCAGCGAG |  |
| pGEX-PHYB-EcoRI-F | GGATCCCCGGAATTCATGGTTTCCGGAGTCGG | pGEX-4T-1-phyB |
| pGEX-PHYB-XhoI-R-3516bp-stop | ATGCGGCCGCTCGAGCTAATATGGCATCATCAGCATCA |  |
| pMal-c2x-PHYB-EcoRI-F | AGGATTTCAGAATTCATGGTTTCCGGAGTCGG | pMAL-c2x-phyB |
| pMal-c2x-PHYB-SalI-R-3516bp-stop | TGCCTGCAGGTCGACCTAATATGGCATCATCAGCATCA |  |
| pHis TF-PHYB-KpnI-F- | CGGGGTACCATGGTTTCCGGAGTCGG | pCold TF-phyB |
| pHis TF-PHYB-Xbal-R- 3516bp stop | TGCTCTAGACTAATATGGCATCATCAGCATCA |  |
| pGEX-SPA1-BamHI-F | GGGCCCCTGGGATCCATGCCTGTTATGGAAAGAGTAGCT | pGEX-4T-1-SPA1 |
| pGEX-SPA1-SalI-R-3087bp | GCCGCTCGAGTCGACAACAAGTTTTAGTAGCTTCATGTTTCC |  |
| pMAL-c2x-SPA1-BamHI-F | CCGGAATTCGGATCCATGCCTGTTATGGAAAGAGTAGCT | pMBP-SPA1 |
| pMAL-c2x-SPA1-SalI-R-3087bp- | TGCCTGCAGGTCGACAACAAGTTTTAGTAGCTTCATGTTTCC |  |
| pET-28a-SPA1-BamHI-F-pET-28a | ATGGGTCGCGGATCCATGCCTGTTATGGAAAGAGTAGCT | pET-28a-SPA1 |
| pET-28a-SPA1-SalI-R-3087bp-pET-28a | CGCAAGCTTGTCGACAACAAGTTTTAGTAGCTTCATGTTTCC |  |
| pET-28a-SPA4-SalI-R-2582bp | CGCAAGCTTGTCGACTACCATCTCCAAAATCTTGATATTG |  |
| pGEX/pMAL-c2x/ pET-28a-BEE1-EcoRI-F | CCGGAATTCATGGCAAATTTCGAGAATCTTTC | pGEX-4T-1-BEE1,  pMAL-c2x-BEE1,  pET-28a-BEE1 |
| pGEX/pMAL-c2x/ pET-28a-BEE1-SalI-R | ACGAGTCGACAAGGGACCATGTTGATAAATGGA |  |
| pGEX/pMAL-c2x/ pET-28a-BEE2-BamHI-F | CGCGGATCCATGGACTTGTCTGTACTTGATAGGCTT | pGEX-4T-1-BEE2,  pMAL-c2x-BEE2,  pET-28a-BEE2 |
| pGEX/pMAL-c2x/ pET-28a-BEE2-SalI-R | ACGCGTCGACCTTGAGGCTGAAGAAATTGGAAAC |  |
| pGEX/pMAL-c2x/ pET-28a-BEE3-EcoRI-F | CCGGAATTCATGGCGAATCTCTCTTCTGATTTT | pGEX-4T-1-BEE3,  pMAL-c2x-BEE3,  pET-28a-BEE3 |
| pGEX/pMAL-c2x/ pET-28a-BEE3-SalI-R | ACGCGTCGACAAGGGTCCACGATGATGAATG |  |
| **Genotyping primers** |  | |
| phyA-211-F | TTATCCACAGGGTTACAGGG | |
| phyA-211-R | GCATTCTCCT TGCATCATCC | |
| phyB-9-F | TTGACTGGTTATGATCGTGTTATGG | |
| phyB-9-R | TGATATATGCAACTGCCATACCG | |
| BEE1-LP | CCCGGAAACTCTCCAGACAGTAGTAACAA | |
| BEE1-RP | CCTTATAACATCCGGGCACCATATCTTGCA | |
| BEE2-LP | GCAGAGGATGAAACAGAGCCAAGCATGAA | |
| BEE2-RP | GGAGGACCTGTGAAGTAAGCCTGAAACTAG | |
| BEE3-LP | CTCTACCTCTTCTGCTCAAGTTTCCATAAA | |
| BEE3-RP | AATCATAGCAAACATCACCAGTCTTACGAG | |
| cop1-6-seq (F) | GGCCACATGAGAAGAACCAGATT | |
| cop1-6-seq (R) | CACAGATTGAAAATCTGCAAGGC | |
| spa1-3-MseI-68bp-F-dCAPS | TAAACATGGTTGATGCACG | |
| spa1-3-MseI-68bp-R-dCAPS | TAAATTTTTTATGTAGTCTTGCTGTT | |
| spa2-1 (823bp) F | GCAGTTAGCTATGCGAAGTTCTTG | |
| spa2-1 (823bp) R | GACCAACTGTAGAACTTTGATTGACC | |
| GABI for spa2-1 | CCCATTTGGACGTGAATGTAGACAC | |
| spa3-1 (1200bp) F | ATGGAAGGTTCTTCAAATTCTAACTCT | |
| spa3-1 (1200bp) R | ACGACTCCTGTTCCTCGATTC | |
| spa4-1 (1100bp) F | GTAACTTTGAAGGCGTGGTTCA | |
| spa4-1 (1100bp) R | CATCTCCAAAATCTTGATATTGCC | |
| LB3 for spa3-1 spa4-1 | TAGCATCTGAATTTCATAACCA | |
| **Real-time qPCR** | | |
| PP2A-RT-F | TATCGGATGACGATTCTTCGTGCAG | |
| PP2A-RT-R | GCTTGGTCGACTATCGGAATGAGAG | |
| ACTIN2-RT-F | TGTGCCAATCTACGAGGGTTT | |
| ACTIN2-RT-R | TTTCCCGCTCTGCTGTTGT | |
| BEE1-RT-F | GGGTTCGGCGAGGGAAAATA | |
| BEE1-RT-R | CTTTGCTCTCTGCATGGAATCA | |
| BEE2-RT-F | CGCAAAACAGTTTCAGGCTTAC | |
| BEE2-RT-R | TTAGCGCCGAGAGATGTGGT | |
| BEE3-RT-F | ATAGCAGAACGGGTTCGACG | |
| BEE3-RT-R | CTCACGTGCCTTTGCCTTCT | |
| BR6OX2-RT-F | CGGTTACCCGCAATCTATG | |
| BR6OX2-RT-R | TAAAGAAAGCAACGAGCCTC | |
| IAA19-RT-F | GGTGACAACTGCGAATACGTTACCA | |
| IAA19-RT-R | CCCGGTAGCATCCGATCTTTTCA | |
| YUC8-RT-F | TGTATGCGGTTGGGTTTACGAGGA | |
| YUC8-RT-R | CCTTGAGCGTTTCGTGGGTTGTTT | |
